# Combining cysteine scanning with chemical labeling to map protein-protein interactions and infer bound structure in an intrinsically disordered region

**DOI:** 10.3389/fmolb.2022.997653

**Published:** 2022-10-07

**Authors:** Shahbaz Ahmed, Gopinath Chattopadhyay, Kavyashree Manjunath, Munmun Bhasin, Neelam Singh, Mubashir Rasool, Sayan Das, Varsha Rana, Neha Khan, Debarghya Mitra, Aparna Asok, Ramandeep Singh, Raghavan Varadarajan

**Affiliations:** ^1^ Molecular Biophysics Unit, Indian Institute of Science, Bangalore, India; ^2^ Institute for Stem Cell Science and Regenerative Medicine, Bangalore, India; ^3^ Tuberculosis Research Laboratory, Translational Health Science and Technology Institute, Faridabad, India

**Keywords:** chemical labeling, protein:protein interaction, functional residues, evolutionary conservation, structure prediction, mutational scanning, deep sequencing

## Abstract

The *Mycobacterium tuberculosis* genome harbours nine toxin-antitoxin (TA) systems of the mazEF family. These consist of two proteins, a toxin and an antitoxin, encoded in an operon. While the toxin has a conserved fold, the antitoxins are structurally diverse and the toxin binding region is typically intrinsically disordered before binding. We describe high throughput methodology for accurate mapping of interfacial residues and apply it to three MazEF complexes. The method involves screening one partner protein against a panel of chemically masked single cysteine mutants of its interacting partner, displayed on the surface of yeast cells. Such libraries have much lower diversity than those generated by saturation mutagenesis, simplifying library generation and data analysis. Further, because of the steric bulk of the masking reagent, labeling of virtually all exposed epitope residues should result in loss of binding, and buried residues are inaccessible to the labeling reagent. The binding residues are deciphered by probing the loss of binding to the labeled cognate partner by flow cytometry. Using this methodology, we have identified the interfacial residues for MazEF3, MazEF6 and MazEF9 TA systems of *M. tuberculosis*. In the case of MazEF9, where a crystal structure was available, there was excellent agreement between our predictions and the crystal structure, superior to those with AlphaFold2. We also report detailed biophysical characterization of the MazEF3 and MazEF9 TA systems and measured the relative affinities between cognate and non-cognate toxin–antitoxin partners in order to probe possible cross-talk between these systems.

## Introduction

There are several methods currently available to determine the structures of proteins and protein:protein complexes such as X ray crystallography, nuclear magnetic resonance (NMR) spectroscopy and cryo-electron microscopy (cryo-EM) (Shi, 2014; Nogales and Scheres, 2015; Kay, 2016; Earl et al., 2017; Jiang and Kalodimos, 2017). While these methods generate a wealth of information, they are time consuming, require a high concentration of purified protein, and are difficult to parallelize. Several *in silico* methods, including, homology modelling, threading, ab initio modelling and machine learning based structure prediction, have been developed to reduce and complement such laborious tasks (Floudas, 2007; Dorn et al., 2014). In the case of homology modelling, there is a threshold of sequence similarity that should be crossed (Dorn et al., 2014), and in the case of threading, it is often difficult to find the best template for a protein with unknown structure, and predict structures for a protein with low sequence identity to the templates (Smith et al., 1997). Ab initio modelling is limited to smaller, monomeric proteins, which are typically ∼100 residues or less (Faver et al., 2011).

Recently, sequence based co-evolution approaches (Göbel et al., 1994; Shindyalov et al., 1994; Pollock et al., 1999) have been used to infer protein structural details (Marks et al., 2012; Sahoo et al., 2015). During the course of evolution, certain specific interaction that influence the structure and function of the protein are maintained either by conservation of these interacting pairs of residues or by correlated mutations at these positions (Godzik and Sander, 1989; Melero et al., 2014). Several methodologies have been developed to identify co-evolving residues within the protein as well as between interacting pairs of proteins and this information act as constraints for model generation of proteins in isolation as well as for modelling of the tertiary structures of protein complexes (Morcos et al., 2011; Kamisetty et al., 2013; Hopf et al., 2014, 2019; Anishchenko et al., 2017; Várnai et al., 2017; Schmidt and Hamacher, 2018; Szurmant and Weigt, 2018). The increase in the number of available protein sequences make this methodology a very convenient tool. However, the effectiveness of this method depends on the occurrence of a large number of homologous sequences, which thereby limits its utility (Kamisetty et al., 2013). Recently machine learning based methods have yielded very promising results (Floudas, 2007; Dorn et al., 2014; Bertoni et al., 2017; Jumper et al., 2021; Tunyasuvunakool et al., 2021; Mirdita et al., 2022). However, the prediction of structures of hetero-oligomeric macromolecular structures, in the absence of structural and sequence based homologs remains challenging (Jumper et al., 2021; Tunyasuvunakool et al., 2021; Mirdita et al., 2022).

Previously, we developed a saturation suppressor mutagenesis based methodology to identify interacting residues in a protein and successfully used this to identify the correct structure of the membrane protein, dgkA (Sahoo et al., 2015). The methodology can be further extended to find interacting residues in a protein complex, first through the identification of interacting residues in the two proteins, subsequently leading to the identification of interacting pairs. There are several known methods to map interacting residues at a protein:protein interface. One such methodology to map functional binding site residues is alanine scanning mutagenesis (Cunningham and Wells, 1989; Wells, 1991; Weiss et al., 2000). A disadvantage of this method is that mutating a residue to alanine does not always inhibit the binding of the cognate partner. Another commonly used methodology is the chemical modification of protein molecules by covalent conjugation (Paus and Winter 2006; Ivanenkov et al., 2010). A common approach to such modification is solvent-accessible cysteine labeling using thiol-reactive dyes (Frillingos et al., 1998; Javitch et al., 2002). This method affords the site-specific labeling of a protein at a unique engineered (or native) surface cysteine. Maleimide is one of the most common reactive groups for cysteine coupling as the coupling reaction is highly specific and efficient.

In the present study, we outline a rapid and efficient method for accurate mapping of protein:protein interactions in the MazEF3, MazEF6 and MazEF9 TA systems of *Mycobacterium tuberculosis*. To identify the interacting residues, we used our previously described, cysteine scanning mutagenesis coupled with fluorescence-activated cell sorting (FACS) methodology (Najar et al., 2017, 2018). This method involves screening a panel of purified cognate proteins or peptides (toxin/antitoxin) against a panel of chemically masked single cysteine mutants of the interacting partner displayed on the surface of yeast cells. Such libraries have much lower diversity than those generated by saturation mutagenesis, simplifying library generation and data analysis. Further, because of the steric bulk of the masking reagent, labeling of virtually all exposed epitope residues should result in loss of binding and buried residues should be inaccessible to the labeling reagent. The binding residues are deciphered by probing the loss of binding of labeled surface displayed protein with its cognate partner by flow cytometry. We have sorted all the libraries together in a pooled format using 1D sort. We also validated our deep sequencing results with a few cysteine mutants both using yeast surface display (YSD) and *in vivo* in *Mycobacterium smegmatis*. The periodicity of mutational sensitivity in the antitoxins was analysed to infer the locations of helical regions in the bound antitoxin. Further, we compared the results obtained from experimental studies with homology modelling and models generated from AlphaFold2 as well as with the recently solved crystal structure of the MazEF9 complex. We observe that for these hetero-oligomeric TA complexes, AlphaFold2 fails to provide reliable models. Our study provides inferences about the putative interacting residues in both globular toxins and intrinsically disordered antitoxins. Thus, our methodology can be extended to other systems where complex structures are either not available or are poorly predicted by the existing modelling programs.

We have also performed detailed functional and biochemical characterisation of the MazEF3 and MazEF9 TA systems from *M. tuberculosis*. In earlier studies, it was shown that growth of *M. bovis* BCG and *M. tuberculosis* were inhibited in a bacteriostatic manner by the inducible expression of the MazF3, F6, and F9 toxins (Tiwari et al., 2015). Here, using nano-differential scanning fluorimetry (nano-DSF) we have measured the relative stabilities of MazE antitoxins, MazF toxins and MazEF complexes. In addition, size exclusion chromatography coupled with multi angle light scattering (SEC-MALS) was performed to characterize the oligomeric status of the free toxins, antitoxins and the TA complexes. We performed YSD and microscale thermophoresis (MST) to determine the relative binding affinities of the toxin with its cognate full-length antitoxin, a peptide containing the C-terminal region of the antitoxin, as well as with the non-cognate full-length antitoxins or peptides containing the C-terminal regions of the antitoxins. The studies reveal significant cross-talk between various members of these TA systems.

## Materials and methods

### Plasmids and host strains

The *mazE* and *mazF* genes were cloned individually under the control of the T7 promoter in the pET-Duet-1 vector for co-expression of the toxin and antitoxin to isolate the toxin-antitoxin (TA) complexes. To purify the individual toxins and antitoxins of the mazEF TA systems, the *mazE* and *mazF* genes were cloned individually in the pET-15b vector.

*Escherichia coli* host strain BL21 (DE3) pLysE was used for expressing the proteins (complexes as well as the toxins and the antitoxins). The *Saccharomyces cerevisiae* strain EBY100 was used for yeast surface display to monitor the binding and expression of the displayed proteins cloned in the yeast surface display vector pPNLS (Chao et al., 2006).

### Cloning of the wild-type and cysteine mutants of *mazE* and *mazF* genes

For cloning of the wild-type (WT) genes, the codon optimized genes of each TA system were PCR amplified from the pET Duet-1 vector. PCR amplified products were gel purified and *in vitro* recombined using Gibson assembly with either pET-15b vector for protein purification, or pPNLS vector for YSD (Gibson et al., 2009). Recombined products were transformed into *E. coli* and plasmid identities were confirmed by Sanger sequencing. The base pair and amino acid sequences of the proteins used in the study along with their molar extinction coefficients are provided as supplementary text.

The WT *mazF6* gene cloned in pET-15b vector was used as a template to introduce the cysteine mutants by PCR as described earlier (Chattopadhyay et al., 2022a) and the amplified products were then PCR purified and *in vitro* recombined using Gibson assembly with either pET-15b or pPNLS. Selected individual cysteine mutants of MazE3, MazE9, MazF9 cloned in pETCON vectors for YSD were synthesised by GenScript (United States).

### Cysteine mutagenesis of *mazE3* and *mazF3*

The MazE3 protein has a cysteine residue at position 98 in the WT sequence and MazF3 protein has two cysteine residues at positions 62 and 71 in the WT sequence. To find whether these are involved in the interaction with MazF3 and MazE3 respectively, the cysteine residues were mutated to alanine. While serine is structurally more similar to cysteine, it is also appreciably more hydrophilic. We therefore replaced WT cysteine residues with alanine instead of serine to prevent the formation of any additional non-covalent interaction with surrounding residues, such as hydrogen bonding through the side chain hydroxyl of serine. The mutations were introduced by three fragment recombination using Gibson assembly (Gibson et al., 2009).

### Expression and purification of the mazEF TA system proteins

The toxins, antitoxins and complexes were purified as described previously (Sharma et al., 2020). The MazEF3 and MazEF9 TA complexes were co-purified, as the toxins had an N-terminal 6x-His tag. The proteins MazE3, MazF3, MazE9 and MazF9 were purified from the pET-15b vector. All the individually expressed toxins and antitoxins have an N-terminal 6x-His tag and a C-terminal 3x-FLAG tag. Ni-NTA affinity purification chromatography was used for purification of complexes as well as individual toxins and antitoxins. Briefly, cultures were grown in terrific broth (TB) media, induced with 1.0 mM IPTG at an OD of 0.6 for 5 h at 37°C for MazE-MazF (His)_6_ complex expression, for 16 h at 20°C for the (His)_6_MazE (FLAG)_3_ antitoxin expression, and for 7 h at 20°C for the (His)_6_MazF(FLAG)_3_ toxin expression. Cells were harvested by centrifugation (1800g, 20 min, 4°C). The pellet was resuspended in resuspension buffer pH 8.0 (10 mM HEPES, 100 mM NaCl, 100 mM arginine, 10% glycerol, 5 mM β-ME containing Protease Inhibitor Cocktail Tablet from Roche) and sonicated, followed by centrifugation at 25,000g, 30 min, 4°C. The His-tagged proteins as well as the complexes were trapped on Ni-NTA resin by mixing 2 ml of the Ni Sepharose 6 Fast Flow (GE Healthcare) with the supernatant, at 4°C for 4 h. The unbound fraction was removed, and the resin was washed with two column volumes of the wash buffer (10 mM HEPES, 100 mM NaCl, 100 mM arginine, 10% glycerol, 5 mM β-ME, 50 mM imidazole, pH 8.0). The complex was then eluted with elution buffer (10 mM HEPES, 100 mM NaCl, 100 mM arginine, 10% glycerol, 5 mM β-ME, gradient of imidazole (100–900 mM), pH 8.0) in 1 ml fractions. The eluted fractions were subjected to 15% Tricine SDS-PAGE, and the protein concentration was determined by absorbance (A_280_) measurements, using their respective molar extinction coefficients. All the proteins were stored in storage buffer (10 mM HEPES, 100 mM NaCl, 100 mM arginine, 10% glycerol, 5 mM β-ME, 500 mM imidazole, pH 8.0, additional cOmplete™ Protease Inhibitor Cocktail Roche for the antitoxins) at -80°C after concentration. The buffer conditions were optimised for the purification of all *M. tuberculosis* proteins. Removing any of the buffer components have been associated with formation of visible aggregates as discussed previously (Chattopadhyay et al., 2022b). Further, a C-terminal MazE3 peptide (residues 72–106), MazE9 peptide (residues 43–76), synthesized from GeneScript was also used in the study.

### Thermal stability measurement using nanoDSF

Thermal stabilities of MazEF complexes, MazE antitoxins and MazF toxins were measured using nanoDSF (Prometheus NT.48) as described previously (Chattopadhyay and Varadarajan, 2019; Chattopadhyay et al., 2022a). Thermal denaturation experiments were carried out at 10 µM protein concentration in the elution buffer for free toxin and antitoxin, as well as TA complex and the normalised first derivative is plotted as a function of temperature as described previously (Chattopadhyay and Varadarajan, 2019; Chattopadhyay et al., 2022a).

### Oligomeric state analysis of the free toxins, antitoxins and TA complexes by size exclusion chromatography coupled with multi angle light scattering (SEC-MALS)

The MazEF complexes, MazE antitoxins and MazF toxins were eluted on a Superdex-200 analytical gel filtration column (GE Healthcare) equilibrated in the elution buffer (10 mM HEPES, 100 mM NaCl, 100 mM arginine, 500 mM imidazole, pH 8.0) and their profiles were monitored with in-line UV (SHIMADZU), MALS (mini DAWN TREOS, Wyatt Technology Corporation) and refractive index (RI) detectors (WATERS 24614) for molecular weight, aggregation and oligomerization analysis at a flow rate of 500 μl/min. For each measurement, 100 µg of each of the proteins were injected. UV, MALS and RI data were collected at room temperature and analysed using ASTRA™ software (Wyatt Technology) (Sharma et al., 2020).

### Binding studies of MazF toxins to full length MazE antitoxins and C-terminal peptides by microScale thermophoresis (MST)

The purified toxins MazF3 and MazF9 were buffer exchanged with 10 mM HEPES, pH 8.0 to remove the primary amines (present in the storage buffer). The toxins were then labeled using the Monolith™ Protein Labeling Kit RED-NHS (NanoTemper Technologies) according to the manufacturer’s instructions. The labeled toxins, MazF3 and MazF9, were used as targets at a concentration of 200 nM each, and were titrated with different concentrations (ranging from 1 pM to 55 μM) of unlabeled antitoxins (full-length and C-terminal peptide) MazE3 and MazE9 respectively. The measurements were done at LED/excitation power setting 20–80%, and at two MST power settings of medium and high. The data was analysed using MO. Affinity Analysis software (version 2.2.5, NanoTemper Technologies) at different standard MST-off times (Wienken et al., 2010; Jerabek-Willemsen et al., 2011; Seidel et al., 2012). To check the presence of any crosstalk between different members of *M. tuberculosis* toxins and antitoxins, the binding of the labeled MazF9 toxin was also probed with different concentrations of the unlabeled full-length antitoxin MazE6 and the peptide containing the C-terminal region of the antitoxin MazE3 (ranging from 1 pM to 5 μM).

### MSA and conservation score calculation of *M. tuberculosis* Maz toxins and antitoxins

The multiple sequence alignment of *M. tuberculosis* toxins MazF1-MazF9 and antitoxins MazE1-MazE9 were carried out using Clustal Omega (Sievers et al., 2011). The MSA generated by Clustal Omega was further used as an input to calculate the evolutionary conservation score from the online server ConSurf (Ashkenazy et al., 2016).

### *M. tuberculosis* MazEF complex structure prediction using AlphaFold2

AlphaFold2, a neural network-based deep learning method (Jumper et al., 2021), was used for the TA complex structure prediction. For prediction of complex structures, the input sequence was provided with the known stoichiometry T_2_A_2_T_2_ and the AlphaFold2-multimer-v2 model type was used. The mmseq2 mode was used for multiple sequence alignment (MSA) and Amber force field was further used for relaxation of the predicted models. The predictions were run on ColabFold (Mirdita et al., 2022).

### Modelling of MazEF3, MazEF6 and MazEF9 complexes and identification of putative interacting residues

Models previously generated for MazEF3, MazEF6 and MazEF9 complexes using homology modelling were used in this study (Tandon et al., 2020). The template for homology modelling was identified from already available structures of MazE and MazF from other organisms based on identity with the query protein (Supplementary Table S1) (Kamada et al., 2003; Simanshu et al., 2013). Additionally, sequence homologs were extracted using BLASTP and aligned with each other, guided by the structure of the template. Apart from homology modelling based on the best template, the query toxins and antitoxins were also aligned with their homologs to find conserved residues and then compared with the available crystal structure data to increase the confidence of predicted toxin/antitoxin interfacial residues (Tandon et al., 2020). The interacting residues of the toxin and antitoxin were identified from the surface accessibility calculations of the residues of the toxin and antitoxin in both the free and bound forms, using NACCESS (Hubbard and Thornton, 1993). Parallelly we overlaid the homology modelled MazF toxins with the template MazF toxin from *Bacillus subtilis* (PDB ID: 4ME7), the residues of modelled toxin which were closest (≤5Å) to the interacting residues of the template MazF toxin were predicted to be interacting. All putative interacting residues predicted from either procedure, were mutated to cysteine using inverse PCR (Jain and Varadarajan, 2014). For the antitoxins, we individually mutated each of the last 35 C-terminal residues of each toxin to cysteine since in most available TA complex structures, it is largely the C-terminal half of the antitoxin that is involved in toxin binding. This was later coupled with YSD and chemical labeling (Najar et al., 2017) for the identification of the interacting residues.

### Yeast surface display of MazE antitoxins and MazF toxins

MazE antitoxins and MazF toxins were expressed on the yeast cell surface and the expression was quantitated as described earlier (Chattopadhyay et al., 2022b). For binding, a slightly modified protocol was used, where 10 µM of the cognate partner, having a 3X FLAG tag was incubated with the yeast cells, and the bound protein amount was detected by the anti FLAG antibody (1:300 dilution) and rabbit anti mouse conjugated to Alexa fluor 633 (1:1,600 dilution) as described earlier (Ahmed et al., 2022a).

### Inverse PCR with adjacent non-overlapping primers to generate single cysteine mutants

An inverse PCR based approach with two non-overlapping but adjacent primers, complementary to different strands of the template was used to generate the single cysteine mutants (Jain and Varadarajan, 2014). PCR amplified products at all the positions were pooled, digested by DpnI overnight, followed by gel extraction. The gel extracted products were then phosphorylated and ligated to generate a circular product. The ligation was confirmed by agarose gel electrophoresis. Ligated products were purified by passing through a column and transformed in high efficiency bacterial electrocompetent cells. The pooled library of each *maz* gene was transformed in EBY100 cells and eight colonies from each library were sent for sequencing. In all the libraries, at least seven different cysteine mutants were found which indicated a good diversity.

### Sorting and deep sequencing of cysteine library for the identification of interacting residues

The yeast cells containing libraries were grown and induced for protein expression as explained earlier (Ahmed et al., 2022a). The ranges of dissociation constants of the labeled cysteine libraries of toxin and antitoxin for binding to their respective cognate partners were measured using yeast surface display. The cells containing cysteine libraries were incubated with 5 mM EZ-Link™ Maleimide-PEG2-Biotin for 1 h at 4°C with shaking, to mask the cysteine residue (10^7^ cells in 500 μl, 5 mM EZ-Link™ Maleimide-PEG2-Biotin). The cells were washed thrice with 200 µl PBS and incubated with the cognate partner. The partner concentrations used were around the concentration of the dissociation constant for the libraries. We sorted the populations based on 1D binding histograms followed by deep sequencing as explained previously (Ahmed et al., 2022b; 2022a) to reconstruct the binding mean fluorescence intensity (MFI) for each mutant in the unlabeled and cysteine masked library. The sorting of MazE and MazF mutants was done using a BD Aria III cell sorter.

In our experimental setup we used an agglutinin-based system to display our mutants. The proteins were fused to the C-terminal Aga2p, and Aga2p is fused to the Aga1P through the disulphide linkages. It is difficult to confirm if the cysteine residues were reduced because the addition of reducing agents will strip the displayed proteins from the surface. In one of our previous study, where we displayed the CcdB cysteine mutants on the yeast cell surface and binding was probed with the GyrA14 protein. The binding was reduced only in the case of CcdB-Gyrase interfacial mutants when cysteine residues were masked (Najar et al., 2017). The residues adjacent to the active site showed no loss in binding upon masking, indicating that the surface exposed cysteine residues were in the reduced form. In the present study, we found several positions where cysteine mutation did not affect the binding, however, upon making cysteine residue the binding was reduced. Further we used a CcdB M32C mutant as a labeling control which loses binding to GyrA14 only after labeling with Biotin-PEG2-Maleimide. This indicates that the cysteine was in the reduced form and could react with the Biotin-PEG2-Maleimide.

### Sample preparation for deep sequencing

Deep sequencing libraries were constructed as described previously (Ahmed et al., 2022a; 2022b). Briefly, the sorted populations were grown on SDCAA agar plates for 48 h, following which the colonies were scraped and plasmid was extracted from the cells. For deep sequencing, the *maz* genes were PCR amplified using primers that bind upstream and downstream of the maz gene sequences. The primers had NNN at the 5’ end, followed by multiplex identifier (MID) sequence to identify the DNA molecules from different sorted populations. PCR was done for 15 cycles, and the amplified product was gel extracted. Equal amounts of DNA were pooled from each sorted population, and the QC was performed to analyse the quality of pooled DNA with Bioanalyzer DNA High Sensitivity chip using Bioanalyzer 2,100 (Agilent). The pooled DNA library was generated using TruSeq™ DNA PCR-Free kit from Illumina and the sequencing was done on an Illumina HiSeq 2,500 platform at Macrogen, South Korea.

### Analysis of deep sequencing data

Sequencing was performed using the Illumina HiSeq 2,500 platform with paired end reads at Macrogen, Korea. The maximum read length that can be obtained from this platform is 2*250 bases from a paired end read. Deep mutational scanning (DMS) data for the *mazE* and *mazF* mutants obtained from the Hiseq 2,500 platform was processed using a slightly modified version of an already existing in-house protocol (https://github.com/skshrutikhare/cys_library_analysis) as described previously (Ahmed et al., 2022a; 2022b). Briefly, the methodology consists of the following steps: assembling the paired end reads, quality filtering, binning, alignment and mutant identification. Paired end reads were first assembled using the PEAR v0.9.6 (Paired-End Read Merger) tool (Zhang et al., 2014) followed by a “quality filtering” step which involves the deletion of terminal “NNN” residues in the reads and removal of reads not containing the relevant MID and/or primers along with the reads having mismatched MIDs. Finally, only those reads having bases with Phred score ≥20 are retained. A further filtering is carried out in the binning step, which eliminates all those reads which have incorrectly placed primers, truncated MIDs/primers (due to quality filtering) and shorter/longer sequences than the length of the wild type sequences. The remaining reads were binned according to the respective MIDs. In the alignment step, reads were aligned with the wild type *mazE* and *mazF* sequence using the Water v6.4.0.0 program (Smith and Waterman, 1981) and reformatted. The default values of all parameters, except the gap opening penalty which was changed to 20, were used. In the final step of “substitution”, reads were classified based on insertions, deletions and substitutions (single, double mutants etc).

### MFI reconstruction from 1D binding histograms

In the case of sorting from 1D binding histograms, the binding MFI of each mutant in both labeled and unlabeled libraries was estimated as explained earlier (Ahmed et al., 2022a). Briefly, reads for each mutant were normalized across different bins individually (Equation 1), and the fraction of each mutant (*Xi*) distributed amongst the different bins was calculated (Equation 2) as given above. The reconstructed MFI for an individual mutant was calculated by the summation of the product, obtained upon multiplying the fraction (*Xi*) of the mutant in a particular bin (i) with MFI of the corresponding bin obtained from the FACS experiment (*Fi*), across the various bins populated by the respective mutant (Eq. 3). The MFI was calculated at a stringency of 100 reads (the minimum value of the sum of the number of reads in all gates combined) (Ahmed et al., 2022a; 2022b). Mutants with a total read number greater than the stringency value were considered for the analysis.

$$\text{Normalized}\;\text{read}\;\text{of}\;\text{mutant}\;\text{in}\;\text{bin}\;i\left(Ni\right)=\frac{Number\;of\;reads\;of\;mutant\;i\;\mathrm{in}\;bin\;i}{\sum Reads\;\mathrm{in}\;bin\;i}$$ (1)

$$\text{Fraction}\;\text{of}\;\text{mutant}\;\text{in}\;\text{each}\;\text{gate}\;\left(Xi\right)=\frac{Ni}{\sum_{1}^{n}Ni}$$ (2)

$$\text{Reconstructed}\;\text{MFI}=\sum_{1}^{n}Fi*Xi$$ (3)

Ratio of depletion of a particular mutant (Unlab/Lab),

$${MFI}_{depletion}^{Mutant}=\frac{{MFI}_{unlabeled}}{{MFI}_{labeled}}$$ (4)

Finally, normalization was done with the WT ratio of depletion as given below:

$$\text{Normalised}\;\text{Fold}\;\text{Change}\;\text{of}\;\text{depletion}=\frac{{\mathrm{MFI}}_{\mathrm{depletion}}^{\mathrm{Mutant}}}{{\mathrm{MFI}}_{\mathrm{depletion}}^{\mathrm{WT}}}$$ (5)

where ${MFI}_{depletion}^{WT}$ is ∼1 as expected.

A log_2_ (fold change) depletion value was finally used for analysis.

### Prediction of helical structural features from mutational data

The MFI^bind^ values for cysteine mutants were averaged over a window of seven residues for MazE3, MazE6 and MazE9 to obtain MFI^avg^ which was then subtracted from the MFI^bind^ values to obtain the corrected cysteine mutational scores. These values were fitted to a simple sinusoidal curve, y = a sin (2π x/b + c), where *π* = 3.14, a = amplitude, b = periodicity and c = phase. For the few residues for which we have no Cysteine mutational data, we have used WT values.

### Analysis of expression and binding of the identified MazE and MazF cysteine mutants on the yeast cell surface

The individual cysteine mutants identified from deep sequencing were transformed into *S. cerevisiae* EBY100 cells as explained earlier (Ahmed et al., 2022a; Chattopadhyay et al., 2022b). The FACS sample preparation and the estimation of expression and binding on the yeast cell surface of the transformed MazE and MazF cysteine mutants were carried out in a similar manner as described earlier (Chattopadhyay et al., 2022b).

### Biophysical characterisation of the MazF6 cysteine mutants

A few of the individual cysteine mutants identified from deep sequencing and validated individually on YSD of the MazF6 toxin were cloned in pET-15b vector and transformed into *Escherichia coli* host strain BL21 (DE3) for protein expression and purification. The protein purification was carried out as described earlier (Sharma et al., 2020). 10 µM of each of the purified proteins was then subjected to thermal denaturation experiments using nanoDSF (Prometheus NT.48) as described previously (Chattopadhyay and Varadarajan, 2019). The oligomeric state of the purified toxins was also analysed by SEC-MALS as described in earlier (Sharma et al., 2020). Briefly, 100 µg of each of the proteins was injected for each measurement and UV, MALS and RI data were collected at room temperature and analysed using ASTRA™ software (Wyatt Technology) as described previously (Sharma et al., 2020).

### *In vivo* activity of the MazE and MazF cysteine mutants

For overexpression studies in *Mycobacterium smegmatis*, the wild type or mutant MazF3 and MazF9 was cloned in an anhydrotetracycline based integrative expression vector (Agarwal et al., 2018). The wild type or mutant antitoxin MazE3 and MazE9 were cloned into an episomal acetamide inducible vector, pLam 12. For growth inhibition studies, the expression of toxin and antitoxin was induced in early-log phase cultures of recombinant *M. smegmatis* strains by the addition of 50 ng/ml anhydrotetracycline (for toxins) or 0.2% acetamide (for antitoxins). The growth of various strains was determined by measuring OD_600_ nm at regular intervals.

### Calculation of sensitivity, specificity and accuracy of our methodology and AlphaFold2

To determine the performance of our methodology and AlphaFold2, we compared the interface residues identified from DMS-FACS, predicted from AlphaFold2 model with the MazEF9 crystal structure and calculated the sensitivity, specificity and accuracy as described below:

$$\text{Sensitivity}=\frac{TP}{TP+FN}$$ (6)

$$\text{Specificity}=\frac{TN}{TN+FP}$$ (7)

$$\text{Accuracy}=\frac{TP+TN}{TP+TN+FP+FN}$$ (8)

where TP, TN, FP and FN refer to number of True Positive, True Negative, False Positive and False Negative respectively.

## Results

### TA complexes are more stable than individual toxins and antitoxins, and form higher oligomeric states

The proteins were eluted using a gradient of imidazole (100–900 mM). The final concentrations of MazEF3 and MazEF9 complexes were 2 mg/ml and their corresponding yields were 2 mg/L. The concentrations of purified MazE3, MazE9, MazF3 and MazF9 were 1 mg/ml, 4 mg/ml, 0.8 mg/ml and 3 mg/ml respectively. Their corresponding yields were 1 mg/L, 4 mg/L, 1.6 mg/L and 6 mg/L respectively. MazF3 and MazF9 showed *Escherichia coli* cell lysis upon toxin induction at 37°C. Therefore, in all cases the toxin expression was carried out at low temperatures.

**FIGURE 1:**
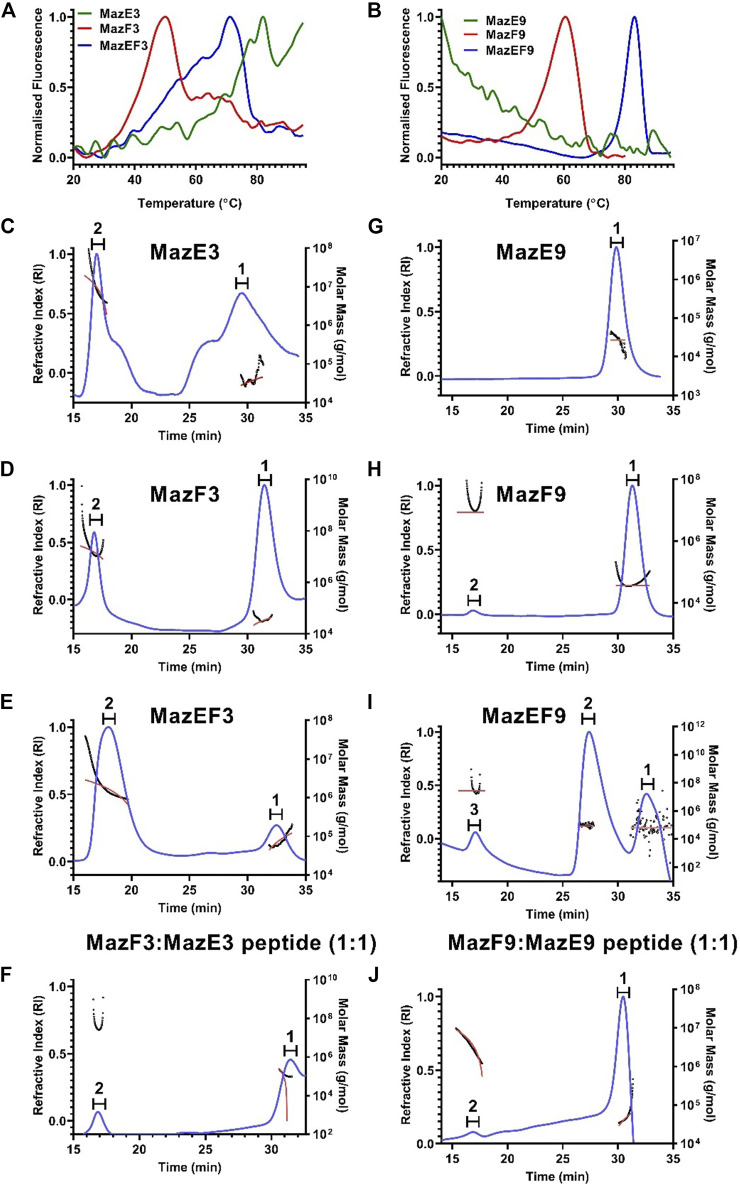
Biophysical characterisation of the MazEF3 and MazEF9 TA systems. **(A,B)** Thermal unfolding profiles of 10 µM of MazEF3, MazEF9 complexes, MazE3, MazE9 antitoxins and MazF3, MazF9 toxins were carried out using nanoDSF. First derivatives of thermal unfolding profile of **(A)** purified MazEF3 complex (blue), MazE3 antitoxin (green) and MazF3 toxin (red), and **(B)** purified MazEF9 complex (blue), MazE9 antitoxin (green) and MazF9 toxin (red). **(C–J)** Oligomeric stoichiometry analysis of the MazEF3 and MazEF9 TA systems by SEC MALS. Traces for refractive index are shown in blue. The molar mass and fits of all traces are plotted as a function of elution time as approximately horizontal red and black lines, respectively. The peaks analysed for molecular weight determination are numbered on top of each graph. The oligomerization status of **(C)** antitoxin MazE3, **(D)** toxin MazF3, **(E)** MazEF3 complex, **(F)** *in vitro* assembled MazEF3 complex with excess MazE3 C-terminal peptide (MazF3:MazE3 peptide:1:2), **(G)** antitoxin MazE9, **(H)** toxin MazF9, **(I)** MazEF9 complex, **(J)** *in vitro* assembled MazEF9 complex with excess MazE9 C-terminal peptide (MazF9:MazE9 peptide:1:2) is shown. The molar mass and mass fraction of each of the peaks are listed in Supplementary Table S2.

All the 6x-His-tagged purified proteins and complexes (10 µM) were subjected to thermal denaturation on the nano-DSF platform. The unfolding was monitored using intrinsic fluorescence of tryptophan and tyrosine residues as a function of temperature, and the apparent T_m_ was calculated (Figures 1A,B). In the cases of the antitoxins MazE3 and MazE9, there was no transition, which indicated that the proteins were intrinsically disordered. All the studied toxins showed proper thermal transitions and T_m_ values were 47 and 61°C for MazF3 and MazF9 respectively. The thermal stabilities of TA complexes are expectedly higher than that of the free toxins and antitoxins (Figures 1A,B) (Cherny et al., 2005; Nieto et al., 2007; Sahoo et al., 2015). The studied complexes showed T_m_ values of 71 and 83°C for MazEF3 and MazEF9 respectively.

Toxins and antitoxins of the MazEF family are dimeric when present in their free forms (Kamada et al., 2003; Simanshu et al., 2013; Zorzini et al., 2014). It has been observed that when the toxins interact with antitoxins, they form hetero-hexameric structures (T_2_A_2_T_2_) in several cases (Kamada et al., 2003; Simanshu et al., 2013). The oligomeric status of free toxins, antitoxins and TA complexes was determined by SEC-MALS. Approximately 100 μg of each of the individual proteins and complexes were analysed under non-denaturing conditions by SEC-MALS in 10 mM HEPES pH 8.0 buffer (containing 100 mM NaCl, 100 mM arginine and 500 mM imidazole) at room temperature.

For the MazEF3 TA system, two different peaks were observed in the RI trace for the individual samples of the antitoxin MazE3, toxin MazF3, complex MazEF3 and *in vitro* reconstituted complex formed between toxin MazF3 and C-terminal MazE3 antitoxin peptide in excess (Figures 1C–F). The peak 2 of MazE3 and MazF3 represent higher order aggregates (molecular weight 500 kDa approx.), whereas the peak 1 corresponds to their dimeric forms (Figures 1C,D). The additional peaks in case of MazE3 could be higher order cysteine aggregates (Figure 1C). The mass fraction of peak 1 of MazEF3 (Figure 1E) which has a molecular weight of 77.6 kDa, was 14.5% and similar to its homolog MazEF in *E. coli* and *Bacillus subtilis* (Kamada et al., 2003; Simanshu et al., 2013), and this peak corresponds to the hetero-hexameric form of the complex (T_2_A_2_T_2_). The mass fraction of peak 1 of the complex formed between MazE3 peptide and MazF3, which has a molecular weight of 43.8 kDa was 93%, and this peak corresponds to the hetero-tetrameric (AT-TA) form of the complex (Figure 1F). In the case of the MazEF9 TA system, we also observed two different peaks in the RI trace for the antitoxin MazE9, toxin MazF9, complex MazEF9 and *in vitro* reconstituted complex formed between toxin MazF9 and C-terminal MazE9 antitoxin peptide in excess (Figures 1G–J). Peak 1 of MazE9 and MazF9 correspond to the dimeric form of the antitoxin (molecular weight 26.9 kDa) and toxin (molecular weight 36.2 kDa) respectively (Figures 1G,H). For MazEF9, peak 2 showed the highest mass fraction (77.2%) with a molecular weight of 84.7 kDa (Figure 1I), which again corresponds to the hetero-hexameric form of the complex (T_2_A_2_T_2_), which is also similar to its homolog MazEF in *E. coli* and *B. subtilis* (Kamada et al., 2003; Simanshu et al., 2013). The mass fraction of peak 1 of the complex formed between MazE9 peptide and MazF9, which has a molecular weight of 43.2 kDa was 87%, and this peak corresponds to the hetero-tetrameric (AT-TA) form of the complex (Figure 1J). Calculated molecular weights of all the peaks of the MazEF TA systems are shown in Supplementary Table S2.

### MazF toxins are more conserved and likely share a similar structural fold as compared to the corresponding MazE antitoxins

MSA for *M. tuberculosis* MazE antitoxins (MazE1-MazE9) was carried out using Clustal Omega (Supplementary Figure S1A). The percent identity amongst the various *M. tuberculosis* MazE antitoxins is in the range of 4–33% (Supplementary Figure S1B). The MSA was used as an input with MazE1 as the query sequence to calculate the conservation score amongst the various *M. tuberculosis* MazE antitoxins using ConSurf (Supplementary Figure S1C). The *M. tuberculosis* MazE antitoxins in general show low sequence identity and an overall poor conservation amongst themselves. An MSA for *M. tuberculosis* MazF toxins (MazF1-MazF9) was also carried out using Clustal Omega (Supplementary Figure S2A). The percent identity amongst the various *M. tuberculosis* MazF toxins is in the range of 9–57% (Supplementary Figure S2B). The MSA was used as an input with MazF1 as the query sequence to calculate the conservation score for the various *M. tuberculosis* MazF toxins using ConSurf (Supplementary Figure S2C). The *M. tuberculosis* MazF toxins in general showed moderate sequence identity and moderate conservation amongst themselves suggesting they might have a similar fold. Across all the MazF toxin structures available in PDB, the backbone root-mean-square deviation (RMSD) is in the range of 0.53–3.03 Å.

### Significant cross-talk is observed between pairs of non-cognate TA systems

The toxins MazF3 and MazF9 were fluorescently labeled with NT-647-NHS dye. The affinities of the fluorescently labeled toxins to their cognate full-length antitoxins or C-terminal antitoxin peptides were analysed using MST as described earlier (Chattopadhyay et al., 2022b). A fixed concentration of 200 nM of the labeled dimeric toxins was titrated with different concentrations of the unlabeled antitoxins (either full length or the peptide, 1 pM-5 μM). The C-terminal peptide which lacks the dimerizing N-terminal domain will be present as a monomer in the solution and thus the monomeric concentration was used for dissociation constant calculations. For the full length antitoxins, which exist as dimers in solution, the monomeric concentration was used to calculate the dissociation constants for reasons described below. In the case of titration of toxins with the antitoxin peptide, it was assumed that only one peptide will bind to a toxin dimer. To estimate the dissociation constant, it was assumed that the binding of toxins to each protomer of the full length antitoxins is identical and independent. The structures of MazEF TA complexes determined so far are hetero-hexamers, in which each protomer of the dimeric antitoxin binds one toxin dimer (Kamada et al., 2003; Simanshu et al., 2013). It is for this reason that we used the dimeric concentration of the toxin and the monomeric concentration of the antitoxin in affinity calculations. The binding study shows that the toxin MazF3 binds with the antitoxin MazE3 peptide (residues 72–106) with an apparent K_D_ of 299 nM (Supplementary Figure S3A). The apparent high K_D_ may arise due to the aggregation of both the toxin and antitoxin. For the MazEF9 system, it was observed that the toxin MazF9 binds with the full length antitoxin MazE9 with a K_D_ of about 8.9 nM and to the MazE9 peptide (residues 43–76) with a K_D_ of about 5.7 nM (Supplementary Figure S3B,C) using MST. The similar binding affinities in the MazEF9 system, suggest that the C-terminal peptides could be used in place of the full-length antitoxins for further binding assays with the toxins, because of the susceptibility of the full-length antitoxins to degradation by proteases. Labeled MazF9 toxin showed significant binding to its non-cognate antitoxin, MazE3 peptide (K_D_ 200 nM, Supplementary Figure S3D), indicating possible cross-talk between these two TA systems (MazEF3 and MazEF9). However, no such interaction was observed between the non-cognate partners of the toxin MazF9 and full-length antitoxin MazE6 (Supplementary Figure S3E). The overall summary of interactions in the MazEF TA system using MST is shown in Supplementary Figure S3F.

### Pooled cysteine libraries can be used to identify interacting residues

**FIGURE 2:**
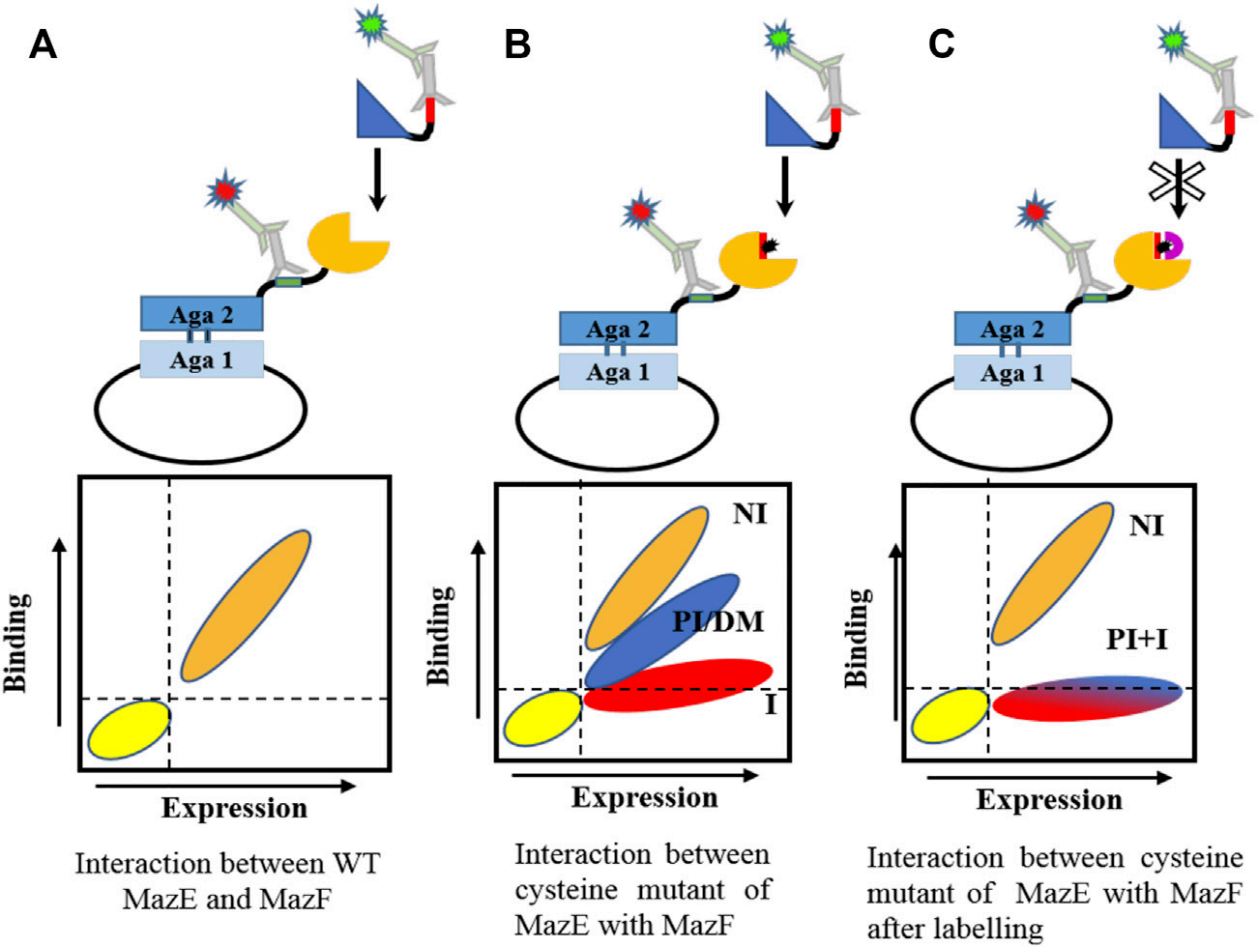
Schematic representation of cysteine scanning mutagenesis methodology. Cysteine mutants of MazF and MazE are introduced at the predicted ligand binding interface. The mutant is displayed as a fusion protein on the surface of yeast cells and its binding to the cognate partner is observed by flow cytometry **(A)** The interaction between WT toxin and antitoxin. **(B)** Introduction of a cysteine mutation in the protein may generate different populations, depending on the location of mutation. Non interacting residues are marked as NI, partially interacting as PI, destabilizing mutations as DM and hot spot residues as I. Mutation at non-interacting sites will not affect binding. However, mutation at the interface or destabilizing mutations will result in a reduced binding wherein the hot-spot residues will lose binding to a very high degree whereas other interfacial residues may or may not show reduced binding. Destabilizing mutations typically occur at buried sites. **(C)** Labeling of cysteine with biotin-PEG2-maleimide should result in loss of binding of all interacting residues. Buried sites are expected to be shielded from labeling.

The interaction between toxins and antitoxins has a very high affinity (De Jonge et al., 2009; Fernández-Bachiller et al., 2016; Kang et al., 2018). The apparent high affinity is likely because of the extensive interaction surface observed between the cognate pairs. As observed for the structures solved for the TA complexes so far, the entire C-terminus and in some cases residues of the N-terminal region of the antitoxin wrap around the toxin and are involved in a number of non-covalent interactions. We measured the affinity of the interaction between WT cognate MazEs and MazFs of MazEF3, MazEF6 and MazEF9 systems and observed a strong interaction between them (Supplementary Figure S3). The cysteine libraries were displayed on the yeast cell surface and their binding was screened against a panel of purified cognate proteins (toxin/antitoxin) before and after labeling (Figure 2).

The MazE3 (C98) and MazF3 (C62, C71) proteins have cysteine residues in the WT sequence. The role of these cysteine residues in binding was identified using cysteine labeling as discussed in the Methods section. Using yeast cells expressing WT MazE3 or MazF3 on the surface, binding to purified MazF3 and MazE3 respectively was probed before and after labeling with 5 mM Biotin-PEG2-maleimide. After labeling, MazE3 WT showed reduced binding (Supplementary Figures S4A,C), suggesting that the cysteine residue in MazE3 is close to the MazE3-MazF3 interface. However, we did not observe any difference in the expression and binding of cells before or after the labeling for MazF3 , suggesting that the cysteine residues in MazF3 are far from the interface of MazF3-MazE3 (Supplementary Figures S4A,C). Since MazF3 C62A-C71A had better expression and binding than WT, it was used for library construction. The modelled MazEF3 and MazEF9 structures were also consistent with this observation. The predicted interacting residues from the homology model for the MazEF3 complex were mutated to cysteine in the background of C98A and C62A-C71A for MazE3 and MazF3 respectively. For the MazEF6 and MazEF9 systems, the predicted interacting residues from the homology models were mutated to cysteine in the background of the WT gene.

The binding of libraries across a range of concentrations of the cognate partner and the apparent K_D_ of the libraries were measured (Supplementary Figure S5). The dissociation constants obtained for MazE3, MazF3, MazE6, MazF6, MazE9 and MazF9 libraries, were in the same range as the dissociation constants obtained for the respective WT proteins. MazE3, MazF3, MazE6, MazF6, MazE9 and MazF9 library had apparent K_D_’s of 1.7, 154, 1.4, 0.13, 1.2 and 6.1 nM respectively (Supplementary Figure S5).

**FIGURE 3:**
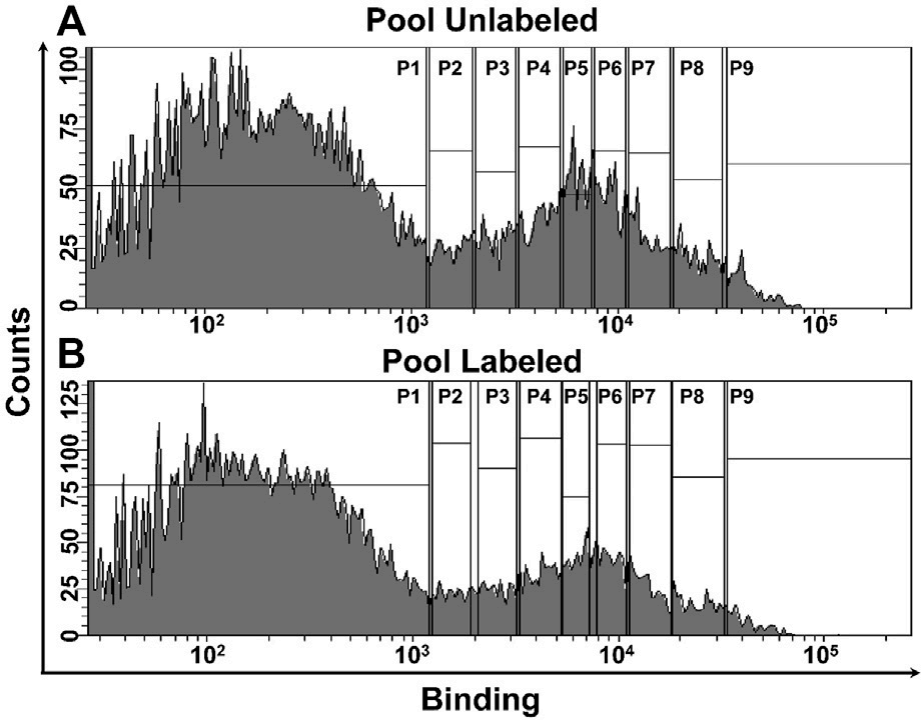
FACS of MazE and MazF libraries. The unlabeled libraries were incubated with the cognate partner (MazF3-200 nM, MazE3- 200 nM; MazF6-375 pM, MazE6- 600 pM; MazF9-10 nM, MazE9- 200 nM). In the case of labeled libraries, the cells expressing cysteine mutants were incubated with 5 mM of labeling reagent, followed by binding with cognate partner at identical concentration used for the unlabeled library. **(A,B)** FACS of the six pooled MazF and MazE libraries. Histogram showing binding of the **(A)** unlabeled and **(B)** labeled libraries. The vertical gates were used to sort different populations based on the binding profiles as described (Ahmed et al., 2022a). Deep sequencing was used to reconstruct the binding MFI of individual members in the unlabeled and labeled libraries (Ahmed et al., 2022a).

**FIGURE 4:**
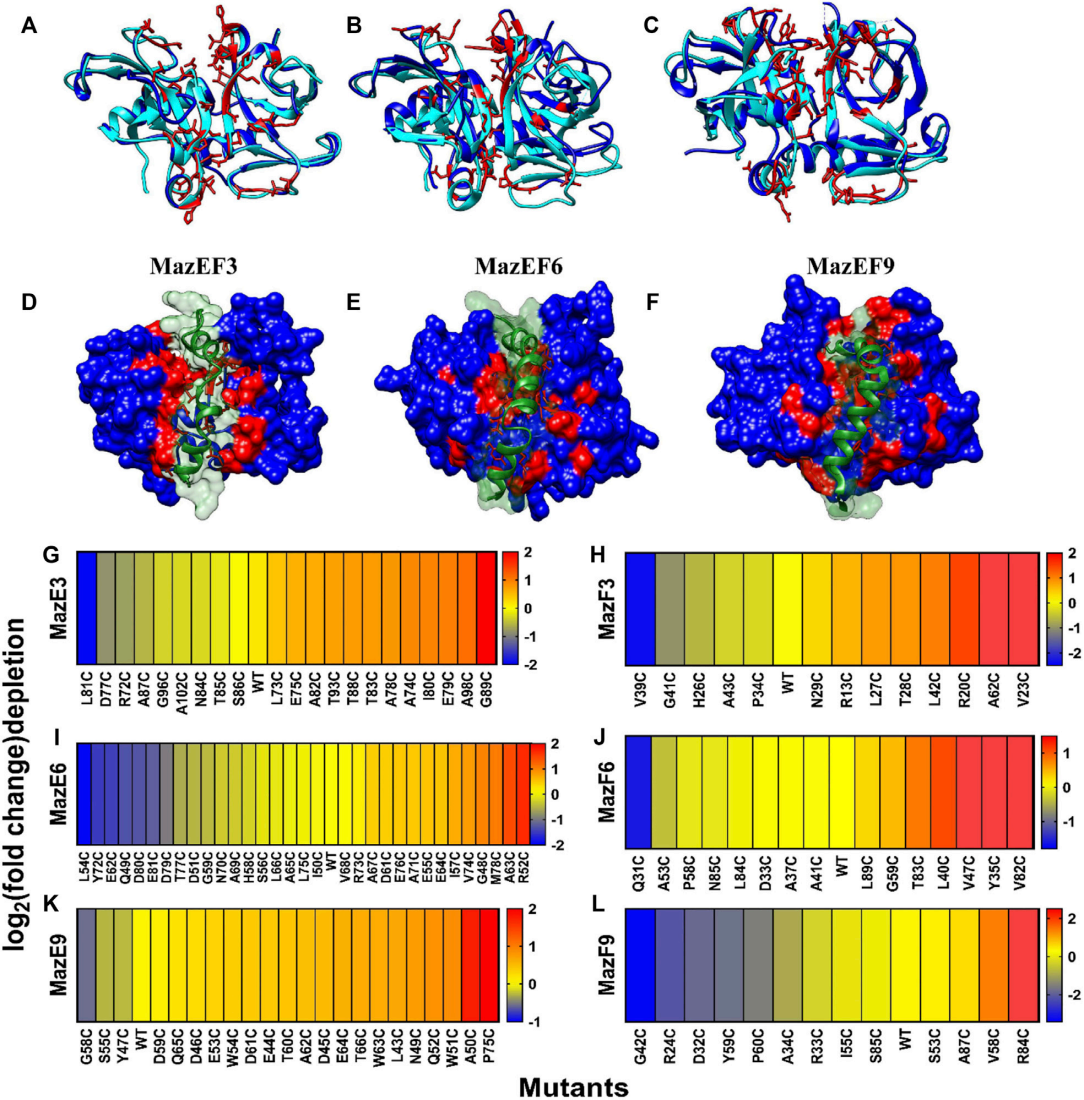
Heat map of fold change of binding after labeling in MazE and MazF libraries from 1D sorts. **(A–F)** Overlay of MazF toxin from *B. subtilis* with the modelled structures of MazF3, MazF6 and MazF9 toxins. *B. subtilis* MazF is in cyan colour **(A)** MazF3 (26%), **(B)** MazF6 (38%) and **(C)** MazF9 (34%) have moderate (%) sequence identity with the *B. subtilis* MazF and are shown in blue. The residues in the modelled toxins which were closest in space to the interacting residues of *B. subtilis* were predicted to be interacting with the cognate antitoxins, are shown in red and were chosen for experimental studies. The bottom panel shows the antitoxin in green which is modelled in complex with the cognate toxin shown in blue, using homology modelling by Modeller v9.14 for **(D)** MazEF3, **(E)** MazEF6 and **(F)** MazEF9 complexes. The predicted interface residues for the toxin are shown in red. Heatmaps showing log_2_ (fold-change) of depletion values after labeling of **(G)** MazE3, **(H)** MazF3, **(I)** MazE6, **(J)** MazF6, **(K)** MazE9 and **(L)** MazF9 cysteine libraries. A stringency of 100 reads in all the gates combined was used as a cut-off for further analysis of mutants. The fold-change cut off for identification of the interacting residues, was determined by k means clustering. Blue to red represents increasing log_2_ (fold change) of depletion values after labeling which is defined as $\log_{2}\left(\frac{{MFI}_{depletion}^{Mutant}}{{MFI}_{depletion}^{WT}}\right)$ . Where, ${MFI}_{depletion}^{Mutant}=\frac{{MFI}_{unlabeled}}{{MFI}_{labeled}}$ . Red denotes positions where substitutions have the maximum affect whereas yellow denotes the residues where there is no effect upon mutation, similar to WT. Blue denotes residues which show increased binding after labeling.

The cysteine libraries were displayed on the yeast cell surface and sorted based on the level of binding into different bins as described above (Figure 3). The residues selected for cysteine mutagenesis were mapped on the MazEF models and are highlighted in red (Figures 4A–F). The *maz* genes from the sorted populations were then amplified and sequenced on an Illumina Hiseq 2,500 platform.

In the case of sorting from a one dimensional binding histogram, we reconstructed the binding MFI of each cysteine mutant from the labeled and unlabeled conditions (Figure 4G-L). A stringency of 100 reads in all the gates combined was used as a cut-off for further analysis of mutants. We observed varying levels of binding upon mutation even in the absence of labeling, which ranged from complete to no loss of binding (Figure 4G-L). The residues which showed ≥15% reduction in binding upon mutation were classified as interacting residues. A second class of interacting residues was also identified as those which showed further reduction in binding upon labeling. The residues which had unlabeled to labeled binding ratio ≥1.2 were considered in this secondary category for all the libraries (Figure 4G-L, Supplementary Tables S3). The cut-off was determined by statistical k-means clustering as described previously (Chandra et al., 2021).

### Homology and AlphaFold2 models were only partially consistent with the experimental data

Due to lesser homologs for the MazEF complexes, we initially compared the crystal structures of the MazF toxins with AlphaFold2 predictions. The analysis was carried out between available MazF toxin structures with their respective models. The backbone RMSD calculated between the predicted and solved crystal MazF toxin structures is in the range of 0.38–1.4 Å. Since the predicted toxin structures were in agreement with the corresponding crystal structures, we proceeded ahead with the prediction of MazEF complex structures by AlphaFold2.

Models of the TA complexes were generated using homology modelling and AlphaFold2multimer model-type (Tandon et al., 2020; Mirdita et al., 2022). To ascertain the predicted interacting residues of the toxin and antitoxin, the surface accessibilities of the residues of the toxin and antitoxin in both the free and bound forms in the model structure were calculated using NACCESS (Hubbard and Thornton, 1993). The predicted interface residues for the toxin involved in antitoxin binding were identified using the difference between the solvent accessible surface area of the toxin residues in the free form and antitoxin-bound form, (ΔASA cut-off ≥1 Å^2^). The interacting residues were also identified experimentally using cysteine labeling and FACS coupled to deep sequencing.

**FIGURE 5:**
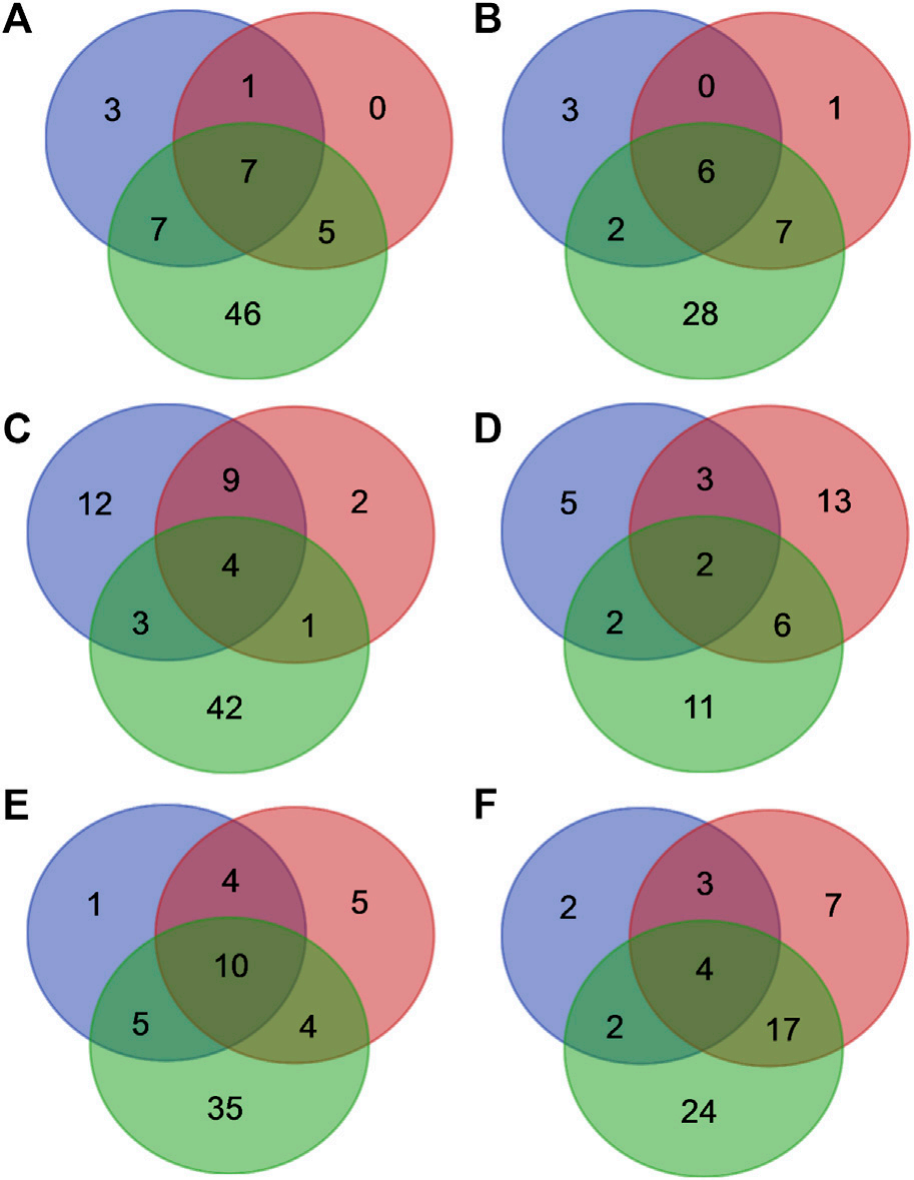
Comparison between interface residues predicted from deep sequencing data, homology models and AlphaFold2 models for the MazEF systems. Venn diagram showing the overlap between the residues predicted to be a part of the interface from the deep sequencing data (blue circle), homology (red circle) and AlphaFold2 (green circle) models of **(A)** MazE3, **(B)** MazF3, **(C)** MazE6, **(D)** MazF6, **(E)** MazE9 and **(F)** MazF9.

Out of twenty-one and thirty-five individual mutants selected for the experimental MazF3 and MazE3 library studies respectively, data for only 16 mutants of MazF3 and 21 mutants of MazE3 were analysed after deep sequencing, as the remaining mutants had very low reads numbers and so they were omitted from the analysis (Figures 5A,B, Supplementary Figures S6A–C, Supplementary Tables S4,S5). The interacting residues obtained from homology model and AlphaFold2 models were mapped onto the model MazEF3 complex (Supplementary Figures S6A-C). A small subset of 6 residues for the MazF3 toxin and 8 residues for the MazE3 antitoxin was found to be present in both the experimentally identified positions and residues identified from homology models (Figures 5A,B). However, other residues which were predicted to be at the interface according to the models did not show any difference in binding upon mutation and/or labeling compared to the WT (Supplementary Tables S4,S5). Additionally, we found reduced binding for some of the mutants, which according to the model were not a part of the predicted set of the interacting residues (Supplementary Tables S4,S5). The AlphaFold2 predictions showed an overlap of 8 and 14 residues for MazF3 toxin and MazE3 antitoxin respectively (Figures 5A,B). Though the overlap between the experimental results and AlphaFold2 predictions was higher than the homology models, the number of false positives was also much higher for AlphaFold2 predicted complex structures (Figures 5A,B).

For the MazEF6 system, out of twenty-one and thirty-five individual mutants selected for the experimental MazF6 and MazE6 library studies respectively, data for only 15 mutants of MazF6 and 33 mutants of MazE6 were analysed after deep sequencing, whereas the remaining mutants had very low reads, and were therefore omitted from the analysis (Figures 5C,D, Supplementary Figures S6D-F, Supplementary Tables S6,S7). As with the MazEF3 system, AlphaFold2 predictions had a large number of false positives for the MazEF6 system as well (Supplementary Tables S6,S7).

In the case of the MazF9 and MazE9 libraries, we selected 26 and 35 putative residues respectively as explained in the previous section for experimental studies (Figures 5E,F, Supplementary Figure S6G-I, Supplementary Tables S8,S9). We could analyse only 15 mutants for MazF9 and 23 for MazE9 after deep sequencing of the samples, the remaining mutants had low reads, and hence were omitted from the analysis (Figures 5E,F, Supplementary Figure S6G-I). A small subset of 7 residues for MazF9 and 14 for MazE9 were common between the experimentally studied positions and residues generated by homology modelling (Figures 5E,F). The predicted complexes from AlphaFold2 showed an overlap of 6 residues and 15 residues of MazF9 toxin and MazE9 antitoxin respectively with the experimental results, with a higher fraction of false positive results (Figures 5E,F). There were other residues in the case of MazEF9 system which were at the interface according to the model but did not show any difference in binding upon mutation and/or labeling compared to the WT from experimental data (Supplementary Tables S8,S9). Here also, we found reduced binding for a few mutants, which according to the model were not part of the predicted set of interacting residues (Supplementary Tables S8,S9).

### Interface identification from DMS-cysteine labeling is more accurate than AlphaFold2 for MazEF9

**FIGURE 6:**
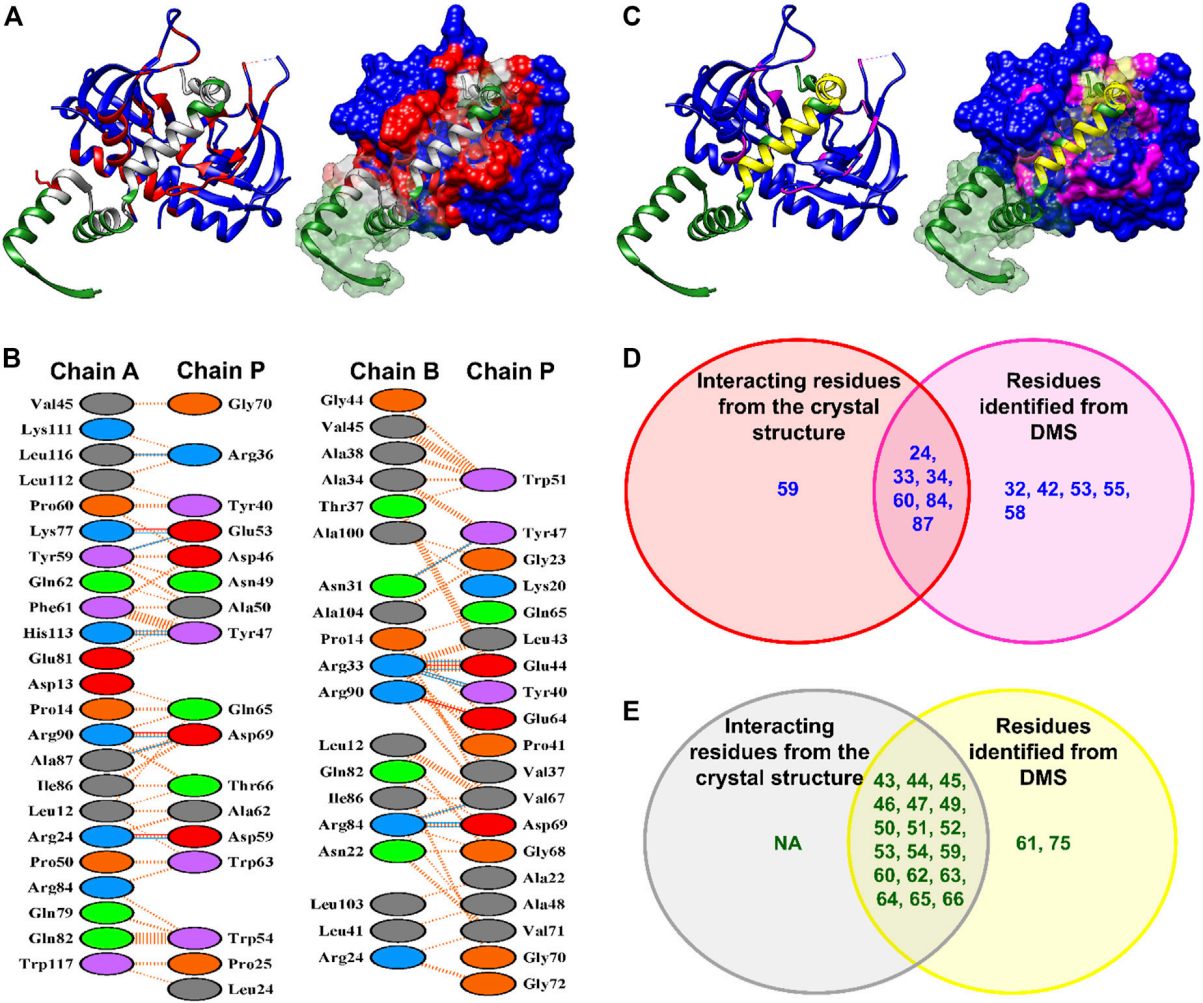
Comparison between interface residues identified from the crystal structure and inferred from deep sequencing data for the MazEF9 system. **(A)** The left panel shows the interface residues determined from the MazEF9 crystal structure. The toxin MazF6 and antitoxin MazE6 are coloured in blue and green respectively. The surface representation of the same is shown in the right panel. The interacting residues are shown in red for MazF9 toxin and in grey for MazE9 antitoxin. Only one monomer of the antitoxin is shown for clarity. **(B)** PDBsum identified interacting residues in MazEF9 structure depicted in Figure **(A)**. Toxin chains (A,B) are each joined by coloured lines to the antitoxin chain (P). Each colour represents a different type of interaction (Salt bridge-Red; Hydrogen bonds-Blue; Non-bonded contacts-Orange **(C)** The left panel shows the experimentally inferred residues from DMS libraries which are highlighted in magenta for MazF9 toxin and yellow for MazE9 antitoxin on the MazEF9 crystal structure. The surface representation of the same is shown in the right panel. **(D–E)** Venn diagram showing the overlap between the interacting residues deciphered from the MazEF9 crystal structure (in red and grey circles) and experimentally verified mutants (in magenta and yellow circles) for **(D)** MazF9 (blue) and **(E)** MazE9 (green). Only residues which were reliably represented in the DMS library after passing the read criteria, are shown in the Venn diagram. ‘NA’ indicates no interacting residues. The seven residues (two from MazE9 library and five from MazF9 library) which were identified from DMS and were not a part of the interface as observed from the crystal structure, are actually in close proximity to the interacting residues. Therefore, mutations to those residues, may have resulted in an apparent decrease in binding. We also could not identify one residue from MazF9 from our cysteine scanning methodology which is actually a part of the MazEF9 interface.

Recently, the MazEF9 crystal structure was solved (Chen et al., 2020). The surface accessibilities for each of the residues of the toxin and antitoxin in both the free and bound forms were calculated using NACCESS (Hubbard and Thornton, 1993). All residues with |ΔASA| ≥ 1 Å^2^ were identified as interface residues. The interacting residues obtained from the ΔASA calculation were mapped on the MazEF9 complex crystal structure in red colour for toxin and grey colour for antitoxin (Figure 6A). The output generated by PDBsum from the MazEF9 crystal structure is shown in Figure 6B. The residues identified experimentally from deep sequencing were mapped on the model structure in magenta colour for toxin MazF9 and yellow colour for antitoxin MazE9 (Figure 6C). A subset of 6 residues for MazF9 and 18 for MazE9 were common between the experimentally studied positions and residues obtained from the crystal structure (Figures 6D,E, Supplementary Tables S8,S9). There were other residues in the case of the MazEF9 system which were at the interface according to the crystal structure but were not chosen for cysteine mutagenesis (Figures 6D,E). Due to the poor homology between MazF homologues, most of the interacting residues of the toxin were not selected for the cysteine mutagenesis study. In the case of the antitoxin, from the crystal structure we found interfacial residues in the 20–72 stretch. However, based on previous studies, we had shortlisted only the last 35 residues of the C-terminus of the antitoxin (43–76) as part of the predicted interface for cysteine mutagenesis. We could not analyse 11 mutants for MazF9 and 12 for MazE9 as these mutants had low reads. From the YSD studies we found a few false positives that were not part of the set of interacting residues in the crystal structure (Figures 6D,E, Supplementary Tables S8,S9). Upon analysing these mutants, most of them were found to be in close proximity to residues which are a part of the interface. Some of these cysteine mutants were also found in loop regions and mutations on this loop may have caused aggregation and thus, caused a decrease in the binding signal. One of the mutants, S85, from the crystal structure appeared to be a part of the interface, but did not show any change in the ΔASA. From previous studies also, we have observed that in the case of a few surface residues, that are not part of the interface in the crystal structure, yet mutations at these positions result in decreased binding (Ahmed et al., 2022a).

We calculated the sensitivity and accuracy of our methodology as described in Equation 6 and 8 and we observe that our methodology has a sensitivity of ∼96% and an accuracy of ∼75%, when the cut-off is |ΔASA| ≥ 1 Å^2^ . From the homology modelling, we predicted residues that were not a part of the interface in the MazEF9 crystal structure. However, the total read count of those residues were less than the cut-off used. Therefore, we did not have any true negative mutants, and thus we did not analyse the specificity parameter.

Incorporating residues which were false positives in case of AlphaFold2, and were determined to be true negatives for homology modelling would have helped to determine the robustness of our methodology. However, most of these residues were initially not present in the libraries, and for some of the residues, there very few sequencing reads, therefore these residues were filtered from subsequent analysis. Larger unbiased libraries with higher read coverage would further enhance the sensitivity, specificity and accuracy of prediction of interfacial residues.

Upon comparison of the interface residues predicted from AlphaFold2 with the MazEF9 crystal structure, we observe a partial overlap of 29 and 33 residues of MazE9 and MazF9 respectively (Supplementary Figure S7). Further, AlphaFold2 could not predict 11 and 15 interacting residues of MazE9 and MazF9 respectively as observed from the crystal structure of the complex (Supplementary Figure S7). The AlphaFold2 model also resulted in 25 and 14 false positive residues for MazE9 and MazF9 respectively (Supplementary Figure S7). We also calculated the sensitivity, specificity and accuracy of the MazEF9 model generated by AlphaFold2 as described in Equation 6-8). We observed that MazEF9 model predicted by AlphaFold2 has a sensitivity of ∼71%, specificity of ∼62% and an accuracy of ∼66%, %, when the cut-off is |ΔASA| ≥ 1 Å^2^.

We also evaluated the performance of our methodology and AlphaFold2 by using higher cut-offs of |ΔASA| ≥ 5 Å^2^ and ≥ 10 Å^2^. The increase in cut-offs did not show significant improvement in sensitivity, specificity and accuracy of the results (Table 1). Overall, the results show that cysteine scan DMS experimental data can add considerable value to structure prediction efforts.

**TABLE 1:** Comparison of performance between Cysteine DMS and AlphaFold2 with MazEF9 crystal structure (PDB ID: 6KYT).

	|ΔASA| ≥1 Å^2^		|ΔASA| ≥ 5 Å^2^		|ΔASA| ≥10 Å^2^	
	Cys DMS	AlphaFold2	Cys DMS	AlphaFold2	Cys DMS	AlphaFold2
Sensitivity	96	70	96	71	95	56
Specificity	NA	62	NA	64	NA	70
Accuracy	75	66	63	66	60	65

### Validation of inferences from deep mutational scanning with YSD of individual mutants

To further validate our deep sequencing results, single mutants were generated, and their binding was measured on the yeast cell surface (Supplementary Figure S8). Relative to MazE3-C98A which showed no decrease in binding before and after labeling, we found that A74C, I80C and L91C mutants showed a decrease in the binding signal upon introducing the cysteine mutation and a further decrease upon labeling (Supplementary Figure S8A). Mutants D77C and G89C showed a marginal decrease in binding upon mutation, and a significant decrease in binding after labeling (Supplementary Figure S8A).

For MazF6, as compared to WT MazF6 which showed no decrease in binding before and after labeling, we found that the cysteine mutants, namely, Q31C, D33C, V47C, V82C, L84C and L89C showed a significant decrease in binding upon mutation (Supplementary Figure S8B). Mutants L84C, L89C along with P58C and G59C, showed a reduction in binding upon mutation, but no further reduction was observed upon labeling (Supplementary Figure S8B). Mutants Y35C and T83C did not show much decrease in binding upon mutation but showed significantly reduced binding upon labeling (Supplementary Figure S8B). The cysteine mutant L40C showed increased binding upon labeling for reasons that are unclear. The mutant A53C did not show any change upon mutation and labeling of the cysteine residue in the mutant (Supplementary Figure S8B).

For MazE9, cysteine mutants W51C and D59C showed a drastic decrease in binding upon mutation as compared to WT MazE9 (Supplementary Figure S8C). The MazE9 mutants, namely, E53C, S55C and P75C did not show any change upon mutation and labeling of the cysteine residue. In the case of MazF9, as compared to the WT MazF9, the cysteine mutants R24C, R84C and A87C showed reduction in binding upon mutation but no further reduction upon labeling was observed (Supplementary Figure S8D). The mutant A34C did not show much decrease in binding upon mutation but showed significantly reduced binding upon labeling (Supplementary Figure S8D). Based on the solved crystal structure of the MazF9 toxin, mutation at a non-interacting site, namely the V58C mutant, also showed reduction in binding upon mutation but no further reduction upon labeling was observed (Supplementary Figure S8D). This could be because of the aggregation tendency of the cysteine mutants on the 53–61 loop (Supplementary Figure S8F).

Overall, the individually analysed mutants showed binding profiles similar to that inferred from the deep sequencing of the pooled libraries (Supplementary Figure S8E).

### Local secondary structural features can be predicted from mutational effects in MazE cysteine variants

**FIGURE 7:**
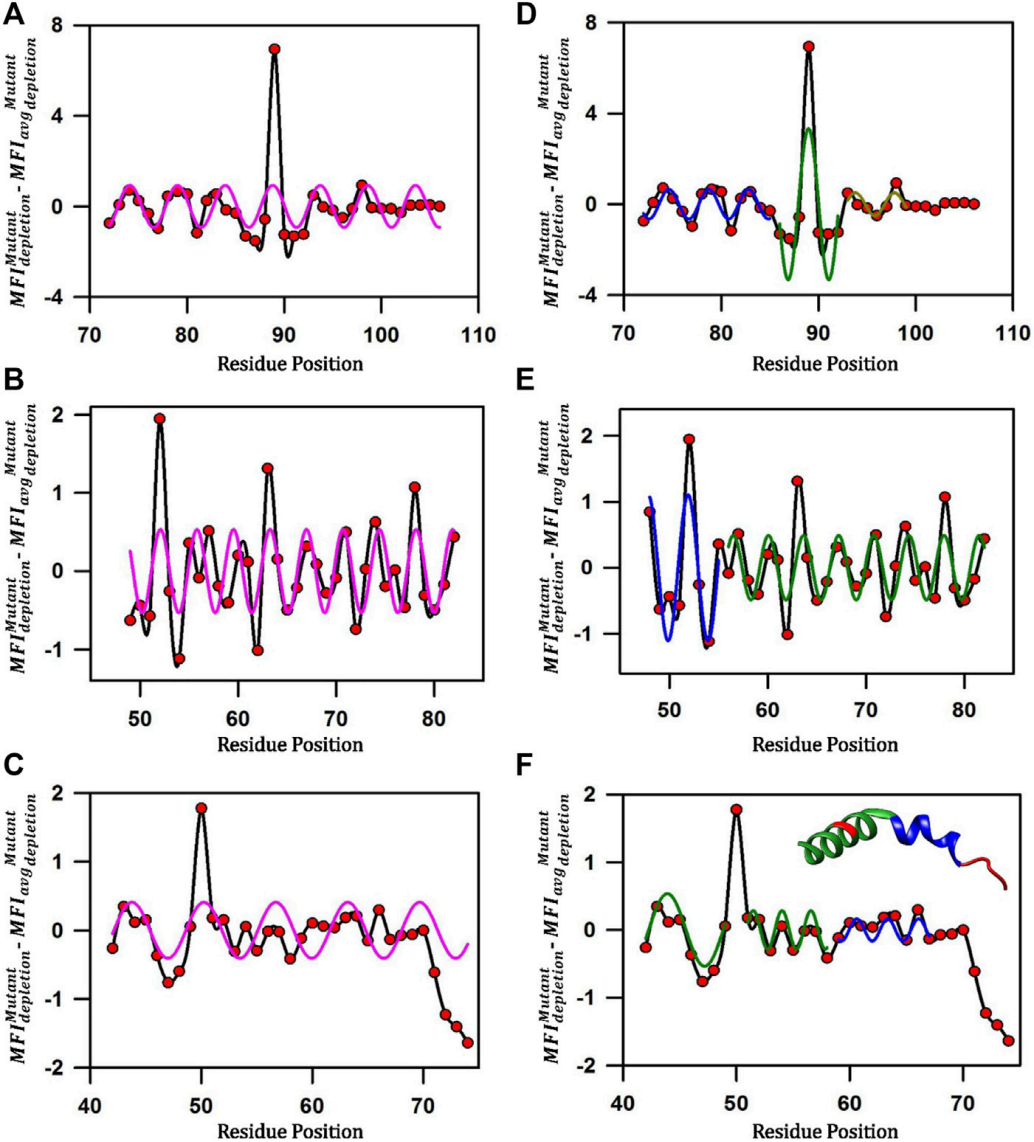
Predicting local structural features attained by disordered MazE antitoxins upon toxin binding. **(A–C)** Corrected mutational scores $\left({MFI}_{depletion}^{Mutant}-{{MFI}_{avg}}_{depletion}^{Mutant}\right)$ of cysteine mutants (red circles) as a function of residue position plotted as a spline curve (black), reveals an oscillating pattern in MazE antitoxin mutational effects. When a single sinusoidal curve (magenta) is fit to the corrected mutational scores of residues 72–106, 48–82, 42–74 for MazE3, MazE6 and MazE9 respectively, the fit is poor. The ${{MFI}_{avg}}_{depletion}^{Mutant}$ is the ${MFI}_{depletion}^{Mutant}$ of cysteine mutants averaged over seven residue windows. **(D–F)** The corrected mutational scores fit to separate sinusoidal curves. **(D)** $\left({MFI}_{depletion}^{Mutant}-{{MFI}_{avg}}_{depletion}^{Mutant}\right)$ for residue stretches 72–85, 86–92 and 93–99 of MazE3 fit to three separate sinusoidal curves. The fits for the 72–85, 86–92 and 93–99 residue stretches are shown in blue, green and dark yellow lines respectively. Residue stretch 100–106 does not fit to a sinusoidal curve. **(E)** For MazE6, the $\left({MFI}_{depletion}^{Mutant}-{{MFI}_{avg}}_{depletion}^{Mutant}\right)$ for residue stretches 48–55 and 56–82 are fit to two separate sinusoidal waves. The fits for 48–55 and 56–82 residue stretches are shown in blue and green lines respectively. (F) $\left({MFI}_{depletion}^{Mutant}-{{MFI}_{avg}}_{depletion}^{Mutant}\right)$ for residue stretches 42–58 (excluding residue 50) and 59–67 of MazE9 fit to two separate sinusoidal waves. The fits for 42–58 and 59–67 residue stretches are shown in green and blue lines respectively. The fourth stretch of residues 68–74 does not fit well to sinusoidal wave. Residue 50 was an outlier and excluded from the fit. In the inset, the MazE9 antitoxin (residues 42–72) from the MazEF9 crystal structure (PDB ID: 6KYT) is shown, with the residue stretches coloured identically to the colours of the fit. Residues 50 and 68–74 are coloured red. The helical regions inferred from the mutational data for MazE9 agree well with those from the crystal structure. The troughs and valleys in the plots correspond to the interacting and non-interacting residues respectively. Both the inferred secondary structure and sites for interaction inferred from the DMS data agree well with the crystal structure. In contrast, PSIPRED (Jones, 1999) predicts helical stretches from residues 39–50, 57–83 and 91–100 in MazE3, residues 9–22, 26–56 and 61–77 in MazE6 and residues 9–21, 27–39, 43–56 and 65–66 in MazE9 respectively.

Mutational scores in the MazE antitoxins across the length of the C-terminal residues under study display an oscillating pattern (Figure 7). To remove the non-uniform region specific contribution to binding, we subtracted from the ${MFI}_{depletion}^{Mutant}$ values, the corresponding values averaged over seven residue windows (Figure 7) as described earlier (Newberry et al., 2020; Chandra et al., 2021). When fitted to a single sinusoidal curve, the corrected cysteine mutational scores $\left({MFI}_{depletion}^{Mutant}-{{MFI}_{avg}}_{depletion}^{Mutant}\right)$ for residues 72–106, 48–82, 42–74 for MazE3, MazE6 and MazE9 respectively shows a poor fit (Figures 7A–C).

For MazE3, the pattern suggested a possible phase change in the wave-like pattern in the mutational effects at residue positions 84–85 and 91–92 (Figure 7A). We therefore fitted residue stretches 72–85, 86–92 and 93–99 to separate sinusoidal waveforms (Figure 7D). The three stretches fitted to individual sinusoidal waves with periodicities of 4.0 ± 0.2, 4.2 ± 0.5 and 4.0 ± 0.5 respectively and with R values of 0.78, 0.77 and 0.74 respectively (Figure 7D). We can therefore infer that the MazE3 residues 72–92 likely form a continuous helix with a distortion around 85–86 and the residues 93–99 form an irregular helical structure (Figure 7D). The stretch from 100–106 forms an irregular structure and did not fit to a sinusoidal curve (periodicity of 7.4 ± 2.1).

In case of MazE6, the pattern suggested a possible phase change in the wave-like pattern in the mutational effects at residue positions 55–56 (Figure 7B). We therefore fitted residue stretches 48–55 and 56–82 to separate sinusoidal waveforms (Figure 7E). The stretches fitted to individual sinusoidal waves with periodicities of 3.8 ± 0.1and 3.6 ± 0.1 respectively and with R values of 0.73, and 0.68 respectively (Figure 7E). We can therefore infer with high confidence that the MazE6 residues 48–55 and 56–82 form a canonical α-helical structure, as the periodicities are close to 3.6 amino acid residues (Figure 7E), which is consistent with a previous study performed using aspartate scanning mutagenesis (Chandra et al., 2021).

For MazE9, residue 50 showed a high depletion value and was excluded from the fit (Figure 7C). We therefore fitted residue stretches 42–58 (excluding residue 50), 59–67 and 68–74 to separate sinusoidal waveforms (Figure 7F). The first three stretches fitted to individual sinusoidal waves with periodicities of 4.0 ± 0.9, 3.5 ± 0.2 and 2.9 ± 0.2 with R values of 0.83, 0.80 and 0.67 respectively (Figure 7F). The third stretch did not fit to a sinusoidal curve (periodicity of 8.3 ± 0.4). We can therefore infer with high confidence that the MazE9 residues 42–58 forms a single helix (Figure 7F). This is consistent with the available MazEF9 crystal structure where residues 43–58 adopts an alpha helical structure and 59–67 forms a non-canonical helix (Figure 7F, inset). The residues 68–72 adopt an irregular structure consistent with the inferences from the cysteine scanning data. Further, from the troughs and valleys of the helices, we can predict the interacting (45,49–52, 54, 60, 63–64, 66) and non-interacting (55–58) residues respectively. The residues identified from the above classification are highly consistent with the observed MazEF9 crystal structure.

Bound conformations of intrinsically disordered protein (IDP) segments are commonly found to form extended helical structures with one face of the helix interacting with the protein partner. Such structural organization in extended helices in IDPs allows facile elucidation of structural features from mutational scanning, using the approach outlined here.

### Most of the purified cysteine mutants were thermally stable and dimeric in solution

It has been shown that the destabilized mutants of protein have lower binding with its ligand on the yeast cell surface (Ahmed et al., 2022a). To confirm that the reduction in binding is due to mutation and masking of interacting residues and not due to the destabilization of mutants, Ni-NTA affinity purification chromatography was used for the purification of a few of the individual cysteine mutants of the MazF6 toxin identified from deep sequencing and validated individually. The proteins were eluted using a gradient of imidazole (100–900 mM) and the eluted fractions were then concentrated and confirmed for the presence of the corresponding proteins by analysing them on 15% Tricine SDS PAGE. All the 6x-His-tagged purified proteins (10 µM) were subjected to thermal denaturation on the nano-DSF platform as described previously (Supplementary Figure S9A) (Chattopadhyay and Varadarajan, 2019). All the studied toxins showed clean thermal transitions and T_m_ values of 64°C, 71°C, 66°C, 86°C, 83°C, 82°C, 66°C, 81 and 79°C for MazF6 WT, D33C, Y35C, V47C, A53C, V82C, T83C, L84C and L89C respectively (Supplementary Figure S9B). The apparent thermal stabilities of the MazF6 cysteine mutants were higher than the MazF6 WT in all replicate measurements. The stability data confirms that the reduction in binding on the yeast cell surface is due to the loss of interaction with its cognate partner, however, we do not understand the underlying reason for the observed higher T_m_ values for several of the cysteine mutants.

We also confirmed that these mutants also maintained their native dimeric state using SEC-MALS under non-reducing conditions. The MazF6 WT and the cysteine mutants Q31C, V47C, A53C, T83C and L89C were eluted as dimers (Supplementary Figure S9C, Supplementary Table S10). From the studies using purified cysteine mutants, we observed that there is a significant enhancement in the apparent thermal stabilities of the mutants, but no mutational effect on the protein oligomeric state, indicating that the loss of binding signal observed in the YSD studies was purely based on the fact that the residues were a part of the interface.

### Phenotypes associated with cysteine mutants in *Mycobacterium smegmatis*

The effect of a few of the cysteine mutants at positions that were predicted to be a part of the interface from the YSD experiment of the MazF3, MazE3, MazE9 and MazF9 systems were studied *in vivo* in *Mycobacterium smegmatis* (Supplementary Figure S10). All the cysteine mutants of the toxins MazF3 and MazF9 showed an inactive phenotype (Supplementary Figure S10A,B). This suggests that there is an overlap between the antitoxin binding site and the active site of the toxin. The other probable reason could be that the cysteine mutants of the toxin are folding defective *in vivo*, thus resulting in no defect in *Mycobacterium* growth. This is unlikely given the results with purified cysteine mutants of the toxins described in the previous sections. When the cysteine mutants of the antitoxins MazE3 and MazE9 were co-expressed with their cognate toxin we observe that only one of the mutants from each MazE3 (D77C) and MazE9 (Y47C) failed to neutralise the toxicity effect of the WT cognate MazF3 and MazF9 toxins (Supplementary Figure S10C,D), indicating these residues are critical in the binding of the toxin. It is hard to precisely compare *in vivo* effect of the cysteine mutants with the *in vitro* YSD experiments because cysteine mutants could not be labeled *in vivo*.

## Discussion

Bacterial toxins regulate growth in response to environmental stress including antibiotic treatment (Hauryliuk et al., 2015). In *E. coli*, there is a single MazF toxin member that cleaves free mRNA to inhibit translation (Zhang et al., 2003). The MazF family is expanded to nine members in *M. tuberculosis,* concurrent with the expansion of different target RNAs including tRNAs and rRNAs (Schifano et al., 2013, 2014, 2016). This expansion suggests that *M. tuberculosis* MazFs may contain different structural elements that recognize diverse RNA substrates.

In this report, we describe preliminary results of a new approach for rapid and reliable mapping of interfacial residues, applied to toxin-antitoxin complexes using a cysteine mutant library displayed on the yeast cell surface. The interacting residues of MazE antitoxins and MazF toxins with cognate partners were first inferred by homology modelling, as well as by overlaying the modelled structure with the template structure. Next, the residues in the modelled structures closest to the interacting residues in the template were designated as putative interacting residues. We subsequently used cysteine scanning methodology coupled to chemical labeling to experimentally identify the interacting residues.

We observed that several putative interacting residues predicted from homology modelling did not show any difference in binding to cognate antitoxin upon labeling for both MazE and MazF proteins. We also observed that several of the putative interacting residues identified from overlaying the model and template showed reduction in binding upon mutation and labeling of the cysteine residue. To reduce the time and effort involved in screening multiple libraries, we pooled multiple libraries as described previously (Ahmed et al., 2022b). Further, we also compared our deep sequencing results with the crystal structure solved for the MazEF9 system. We found that our system is highly sensitive and moderately accurate. Further, deep sequencing data agreed well with the individually analysed mutant data wherein the mutants were isolated using flow cytometry. Since this was the initial application of the methodology to TA systems, we predicted residues from the homology modelling only for the toxins, as the toxin fold is relatively well conserved. For the antitoxins, we did not predict residues from the homology models, rather we mutated the last 35 C-terminal residues based on structural data from other type II TA complexes in which residues from the C-terminal half of the antitoxin are typically involved in toxin binding. Now that the methodology is standardized and validated, in future studies, one would mutate all predicted surface residues for the folded toxin component and all residues for the antitoxin component.

We also inferred local secondary structural features from mutational effects in cysteine variants of MazE antitoxins. In the case of MazE9, using the fitted periodicity of the toxin binding activity of mutants, we predicted an α-helical 42–58 residue stretch, followed by a distorted α-helical 59–67 residue stretch, and a disordered toxin-interacting 68–72 residue stretch. The results obtained are highly consistent with the observed structural secondary features and interfacial residues of MazE9 antitoxin in the MazEF9 crystal structure. The available complex structures of MazEF homologs indicate that all the antitoxin structures are unique and differ significantly from the structural and interfacial features in terms of helical content of the C-terminal domain as well as region specific contribution of N- and C-terminus to cognate toxin binding. Therefore, scanning mutagenesis methods can be employed in deciphering toxin-antitoxin interaction modules and predicting local secondary structures of the antitoxin upon complex formation.

In the past few decades, with the advent of Critical Assessment of protein Structure Prediction (CASP), there have been significant advancements in the field of protein structure determination from sequence information (Moult et al., 1995). Both the global distance test score, a measure of accuracy in the prediction of the protein structure and the average precision in structure prediction have increased from ∼35% in 2006 to ∼90% in 2020 and from 21% in CASP10 to 70% in CASP13 respectively (Schaarschmidt et al., 2018; Shrestha et al., 2019). Advancement in the methods to predict 3D contacts between pairs of residues in a protein termed as contact prediction, is one of the main driving forces for the improved precision and accuracy of the structure predicting tools. In both CASP13 and CASP14 held in 2018 and 2020 respectively, Deep Mind’s AlphaFold and AlphaFold2 have been ranked the highest amongst the protein structure prediction tools (Senior et al., 2020; Jumper et al., 2021). The predictions were claimed to be highly accurate and close to the experimentally determined structures with 95% of the predicted structures having a backbone RMSD of <1Å with the solved experimental structures (Jumper et al., 2021; Tunyasuvunakool et al., 2021). Recently, programs such as SWISS-MODEL (Waterhouse et al., 2018) and RoseTTA fold (Baek et al., 2021) have been developed to predict the structures of proteins as well as complexes. SWISS-MODEL employs homology modelling to build models defined by the target-template alignment followed by quality estimation of the model (Waterhouse et al., 2018). RoseTTA fold uses a three-track neural network with multiple connections between the tracks to inspect the relationship within and between the patterns in protein sequences, distances and coordinates simultaneously (Baek et al., 2021). We also used AlphaFold2 to generate models of the TA complexes used in this study and find the inferences made from the present cysteine scanning approach are much more sensitive and accurate than predictions from AlphaFold2 for the MazEF9 complex (Chen et al., 2020). For the MazEF models, by visual inspection the models did not appear to be properly folded. In the multiple sequence alignments (MSA) generated by AlphaFold2 there are several gaps in the alignments. It is likely that the poor performance of AlphaFold2 in the present case, is because of the limited number of sequence homologs for the three MazEF systems studied here.

In conclusion, we described and validated high-throughput methodology to rapidly identify interacting residues in a protein:protein complex with high efficiency, which can be used for model discrimination and structure prediction in other systems.

## Data Availability

The deep sequencing data discussed in the present study have been deposited in NCBI’s Sequence Read Archive (SRA no: SRR16071134). Illumina sequencing counts for each ccdB double mutant of FACS bins are available at https://github.com/rvaradarajanlab/Cysteine-scanning-mutagenesis. The data relevant to the figures in the paper have been made available within the article and in the Supplementary Material. All unique/stable reagents generated in this study are available from the Lead Contact RV (varadar@iisc.ac.in) without restriction.
