# Supplementary material for: Combining cysteine scanning with chemical labeling to map protein-protein interactions and infer bound structure in an intrinsically disordered region
Source: Front Mol Biosci. 2022 Oct 7;9:997653. doi: 10.3389/fmolb.2022.997653 (PMC9585320; doi:10.3389/fmolb.2022.997653)
Supplement: Supplementary file 1 [file DataSheet1.pdf]

Supplementary text

The nucleotide and amino acid sequences of the proteins used in the study along with their molar extinction coefficients.

MazE3 (Molar extinction coefficient: 12950 M^{-1} cm^{-1}**)**
ATGGGCAGCAGCCATCATCATCATCATCACAGCAGCGGCCTGGTGCCGCGCGGCAGCCA
TATGATGTACCTGCCGTGGGGCGTGGTTCTGGCGGGTGGCGCGAACGGTTTTGGTGCGG
GTGCGTATCAGACCGGCACCATCTGCGAGGTGAGCACCCAAATTGCGGTTCGTCTGCCG
GACGAGATCGTGGCGTTTATTGACGATGAAGTTCGTGGTCAGCATGCGCGTAGCCGTGC
GGCGGTGGTTCTGCGTGCGCTGGAGCGTGAACGTCGTCGTCGTCTGGCGGAGCGTGATG
CGGAAATCCTGGCGACCAACACCAGCGCGACCGGCGACCTGGATACCCTGGCGGGTCA
CTGCGCGCGTACCGCGCTGGACATTGATGCGGCCGCGGGCGCGGGTTCGGGCGATTACA
AAGACCACGACGGTGACTACAAAGACCACGACATTGACTACAAAGATGATGACGACAA
ATAATAA

MGSSHHHHHHSSGLVPRGSHMMYLPWGVVLAGGANGFGAGAYQTGTICEVSTQIAVRLPD
EIVAFIDDEVRGQHARSRAAVVLRALERERRRRLAERDAEILATNTSATGDLDTLAGHCART
ALDIDAAAGAGSGDYKDHDGDYKDHDIDYKDDDDK**

MazF3 (Molar extinction coefficient: 11460 M^{-1} cm^{-1}**)**
ATGGGCAGCAGCCATCATCATCATCATCACAGCAGCGGCCTGGTGCCGCGCGGCAGCCA
TATGATGCGTCCGATCCACATTGCGCAACTGGACAAGGCGCGTCCGGTGCTGATCCTGA
CCCGTGAGGTGGTTCGTCCGCACCTGACCAACGTGACCGTTGCGCCGATTACCACCACC
GTGCGTGGTCTGGCGACCGAAGTGCCGGTTGATGCGGTTAACGGCCTGAACCAGCCGAG
CGTGGTTAGCTGCGACAACACCCAAACCATCCCGGTTTGCGATCTGGGTCGTCAGATTG
GCTACCTGCTGGCGAGCCAGGAACCGGCGCTGGCGGAAGCGATTGGTAACGCGTTCGA
CCTGGATTGGGTGGTTGCGGCGGCCGCGGGCGCGGGTTCGGGCGATTACAAAGACCAC
GACGGTGACTACAAAGACCACGACATTGACTACAAAGATGATGACGACAAATAATAA

MGSSHHHHHHSSGLVPRGSHMMRPIHIAQLDKARPVLILTREVVRPHLTNVTVAPITTTVRG
LATEVPVDAVNGLNQPSVVSCDNTQTIPVCDLGRQIGYLLASQEPALAEAIGNAFDLDWVV
AAAAGAGSGDYKDHDGDYKDHDIDYKDDDDK**

MazE6 (Molar extinction coefficient: 12950 M^{-1} cm^{-1}**)**
ATGGGCAGCAGCCATCATCATCATCATCACAGCAGCGGCCTGGTGCCGCGCGGCAGCCA
TATGATGAAAACCGCGATCAGCCTGCCGGACGAAACCTTCGATCGTGTGAGCCGTCGTG
CGAGCGAGCTGGGTATGAGCCGTAGCGAGTTCTTCACCAAAGCGGCGCAGCGTTACCTG
CACGAGCTGGATGCGCAGCTGCTGACCGGTCAAATCGATCGTGCGCTGGAAAGCATTCA
CGGCACCGATGAGGCGGAAGCGCTGGCGGTGGCGAACGCGTATCGTGTGCTGGAGACG
ATGGACGATGAATGGGCGGCCGCGGGCGCGGGTTCGGGCGATTACAAAGACCACGACG
GTGACTACAAAGACCACGACATTGACTACAAAGATGATGACGACAAATAATAA

MGSSHHHHHHSSGLVPRGSHMMKTAISLPDETFDRVSRRASELGMSRSEFFTKAAQRYLHE
LDAQLLTGQIDRALESIHGTDEAEALAVANAYRVLETMDDEWAAAGAGSGDYKDHDGDY
KDHDIDYKDDDDK**

MazF6 (Molar extinction coefficient: 12950 M^{-1} cm^{-1}**)**
ATGGGCAGCAGCCATCATCATCATCATCACAGCAGCGGCCTGGTGCCGCGCGGCAGCCA
TATGATGGTGATCAGCCGTGCGGAGATTTACTGGGCGGACCTGGGTCCGCCGAGCGGCA
GCCAGCCGGCGAAGCGTCGTCCGGTGCTGGTTATCCAAAGCGATCCGTATAACGCGAGC
CGTCTGGCGACCGTGATTGCGGCGGTTATTACCAGCAACACCGCGCTGGCGGCGATGCC
GGGTAACGTTTTCCTGCCGGCGACCACCACCCGTCTGCCGCGTGACAGCGTGGTTAACG
TGACCGCGATTGTTACCCTGAACAAAACCGACCTGACCGATCGTGTGGGTGAGGTTCCG
GCGAGCCTGATGCACGAAGTGGACCGTGGCCTGCGTCGTGTTCTGGATCTGGCGGCCGC
GGGCGCGGGTTCGGGCGATTACAAAGACCACGACGGTGACTACAAAGACCACGACATT
GACTACAAAGATGATGACGACAAATAATAA

MGSSHHHHHHSSGLVPRGSHMMVISRAEIYWADLGPPSGSQPAKRRPVLVIQSDPYNASRL
ATVIAAVITSNTALAAMPGNVFLPATTTRLPRDSVVNVTAIVTLNKTDLTDRVGEVPASLMH
EVDRGLRRVLDLAAAGAGSGDYKDHDGDYKDHDIDYKDDDDK**

MazE9 (Molar extinction coefficient: 25440 M^{-1} cm^{-1}**)**
ATGGGCAGCAGCCATCATCATCATCATCACAGCAGCGGCCTGGTGCCGCGCGGCAGCCA
TATGATGAAGCTGAGCGTGAGCCTGAGCGACGATGACGTTGCGATCCTGGATGCGTACG
TGAAGCGTGCGGGTCTGCCGAGCCGTAGCGCGGGTCTGCAACACGCGATTCGTGTTCTG
CGTTACCCGACCCTGGAGGATGACTATGCGAACGCGTGGCAAGAATGGAGCGCGGCGG
GCGATACCGATGCGTGGGAGCAAACCGTGGGTGATGGTGTTGGTGATGCGCCGCGTGC
GGCCGCGGGCGCGGGTTCGGGCGATTACAAAGACCACGACGGTGACTACAAAGACCAC
GACATTGACTACAAAGATGATGACGACAAATAATAA

MGSSHHHHHHSSGLVPRGSHMMKLSVSLSDDDVAILDAYVKRAGLPSRSAGLQHAIRVLR
YPTLEDDYANAWQEWSAAGDTDAWEQTVGDGVGDAPRAAAGAGSGDYKDHDGDYKDH
DIDYKDDDDK**

MazF9 (Molar extinction coefficient: 16960 M^{-1} cm^{-1}**)**
ATGGGCAGCAGCCATCATCATCATCATCACAGCAGCGGCCTGGTGCCGCGCGGCAGCCA
TATGATGATGCGTCGTGGTGAAATCTGGCAGGTGGACCTGGACCCGGCGCGTGGCAGCG
AAGCGAACAACCAACGTCCGGCGGTGGTTGTGAGCAACGATCGTGCGAACGCGACCGC
GACCCGTCTGGGTCGTGGCGTTATCACCGTTGTGCCGGTGACCAGCAACATTGCGAAGG
TTTACCCGTTTCAAGTGCTGCTGAGCGCGACCACCACCGGTCTGCAAGTTGATTGCAAA
GCGCAGGCGGAGCAAATCCGTAGCATTGCGACCGAACGTCTGCTGCGTCCGATTGGTCG
TGTGAGCGCGGCGGAGCTGGCGCAGCTGGACGAAGCGCTGAAGCTGCACCTGGATCTG
TGGAGCGCGGCCGCGGGCGCGGGTTCGGGCGATTACAAAGACCACGACGGTGACTACA
AAGACCACGACATTGACTACAAAGATGATGACGACAAATAATAA

MGSSHHHHHHSSGLVPRGSHMMMRRGEIWQVDLDPARGSEANNQRPAVVVSNDRANATA
TRLGRGVITVVPVTSNIAKVYPFQVLLSATTTGLQVDCKAQAEQIRSIATERLLRPIGRVSAA
ELAQLDEALKLHLDLWSAAAGAGSGDYKDHDGDYKDHDIDYKDDDDK**

Supplementary Figures

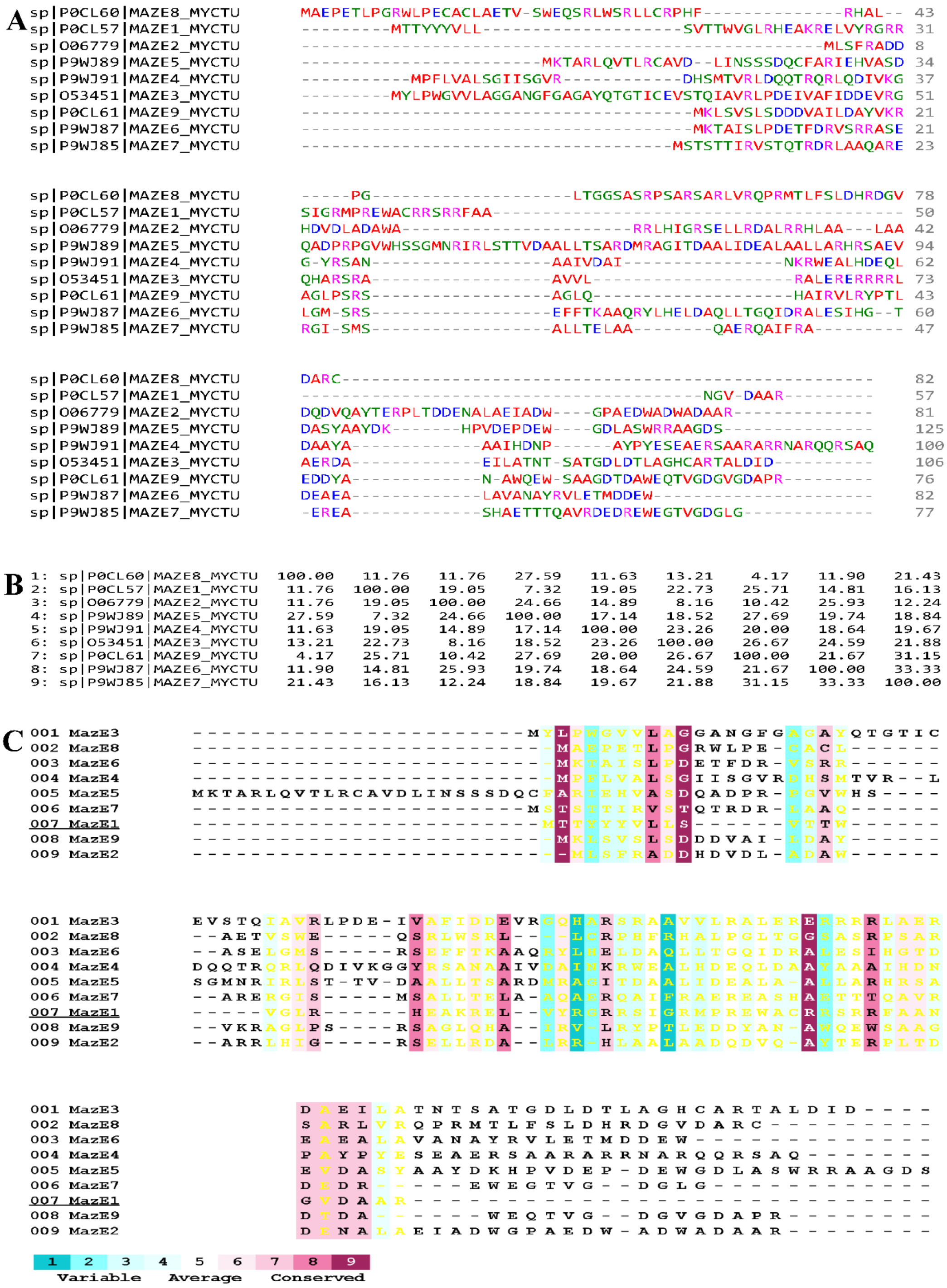

Figure S1 Multiple Sequence Alignment of *M. tuberculosis* MazE homologs. (A) MSA for *Mtb* MazE1-MazE9. (B) Matrix showing the percent identity among the various *M. tuberculosis* MazE antitoxins. (C) Conservation scores of the *Mtb* MazE antitoxins generated by ConSurf. Some of the most conserved residues in case of MazE antitoxins were experimentally identified to be a part of the interface. Some of the N-terminus residues, probably involved in DNA binding are also conserved.

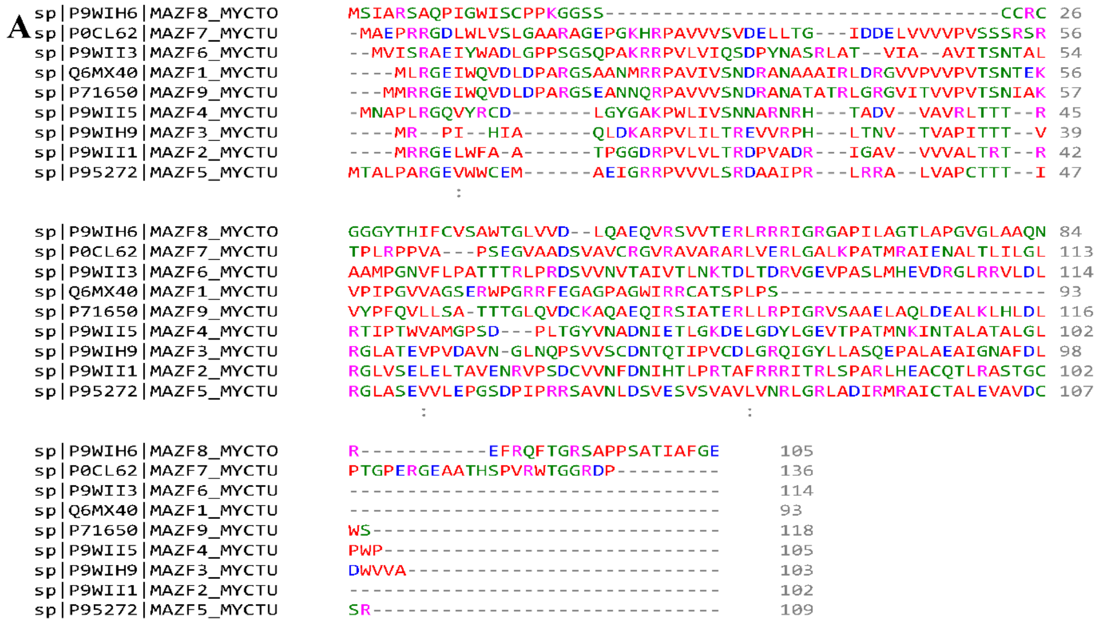

B

		1	2	3	4	5	6	7	8	9
1:	sp|P9WIH6|MAZF8_MYCTO	100.00	16.30	18.29	17.54	30.86	9.72	20.59	11.27	16.88
2:	sp|P0CL62|MAZF7_MYCTU	16.30	100.00	19.44	32.18	31.86	26.47	18.00	27.27	30.48
3:	sp|P9WII3|MAZF6_MYCTU	18.29	19.44	100.00	25.84	29.46	27.84	26.60	30.61	23.76
4:	sp|Q6MX40|MAZF1_MYCTU	17.54	32.18	25.84	100.00	57.61	26.32	16.00	15.19	25.00
5:	sp|P71650|MAZF9_MYCTU	30.86	31.86	29.46	57.61	100.00	19.80	23.23	24.75	21.90
6:	sp|P9WII5|MAZF4_MYCTU	9.72	26.47	27.84	26.32	19.80	100.00	23.47	25.51	27.88
7:	sp|P9WIH9|MAZF3_MYCTU	20.59	18.00	26.60	16.00	23.23	23.47	100.00	30.93	30.00
8:	sp|P9WII1|MAZF2_MYCTU	11.27	27.27	30.61	15.19	24.75	25.51	30.93	100.00	32.35
9:	sp|P95272|MAZF5_MYCTU	16.88	30.48	23.76	25.00	21.90	27.88	30.00	32.35	100.00

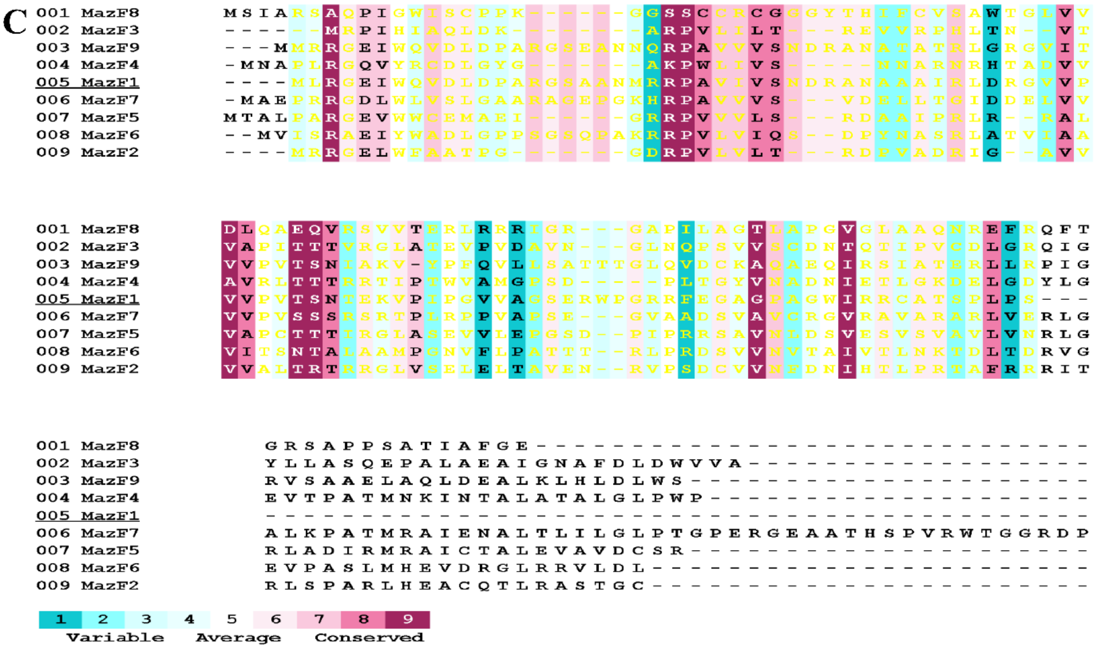

Figure S2 Multiple Sequence Alignment of *M. tuberculosis* MazF homologs. (A) MSA for *Mtb* MazF1-MazF9. (B) Matrix showing the percent identity among the various *Mtb* MazF toxins. (C) Conservation scores of the *M. tuberculosis* MazF toxins generated by ConSurf. Most of the highly conserved residues in case of MazF toxins were not identified as a part of the interface and could be a part of the active site of the toxins.

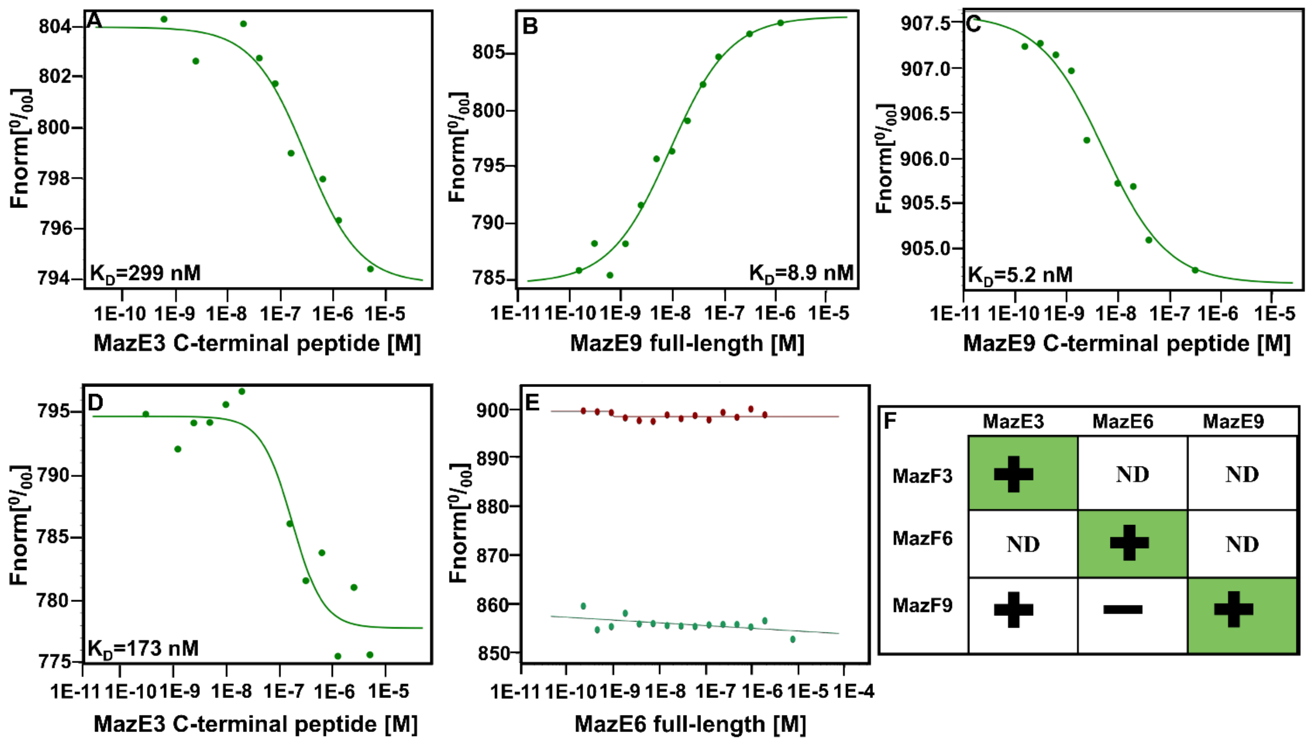

Figure S3 Characterization of interactions of MazF toxins with other cognate and non-cognate antitoxins by MicroScale Thermophoresis. Toxins MazF3 and MazF9 were labeled with Monolith™ Protein Labeling Kit NT-647-NHS dye (NanoTemper Technologies) according to the manufacturer's instructions, used at a concentration of 200 nM and titrated with different concentration ranges (1 pM-5 µM) of (A) MazE3 peptide (residues 72-106) with MazF3 toxin, (B) full-length antitoxin MazE9 and (C) C -terminal peptide of the MazE9 antitoxin (residues 43-76) with MazF9 toxin to determine the K_D. (D-E) 200 nM of labeled toxin MazF9 was titrated with different concentration ranges of (D) MazE3 C-terminal peptide (residues 72-106) and (E) full-length MazE6 antitoxin to probe for possible cross talk. (F) Summary of MazEF toxin-antitoxin interaction as determined by MicroScale Thermophoresis**.** For all the interactions studied *in vitro* by MST, the symbols in the key (+) denotes detectable interaction, (-) denotes no interactions and ND denotes the interaction which was not determined. All studies were carried out in 10 mM HEPES, pH 8.0 and at 27 °C. The normalised fluorescence FNorm [$^0/_{00}$] is plotted as a function of [MazE]. For each capillary (each measuring point), an MST trace is recorded. All traces are then normalised to start at 1000. For

each trace, the FNorm value for the dose - response curve is calculated from Fhot (MST laser on)/Fcold (MST laser off). The K_D values for the interactions were determined employing standard data analysis with MO.Affinity analysis software (5s MST-on time for evaluation) (Wienken et al., 2010; Jerabek-Willemsen et al., 2011; Seidel et al., 2012; Chattopadhyay et al., 2022).

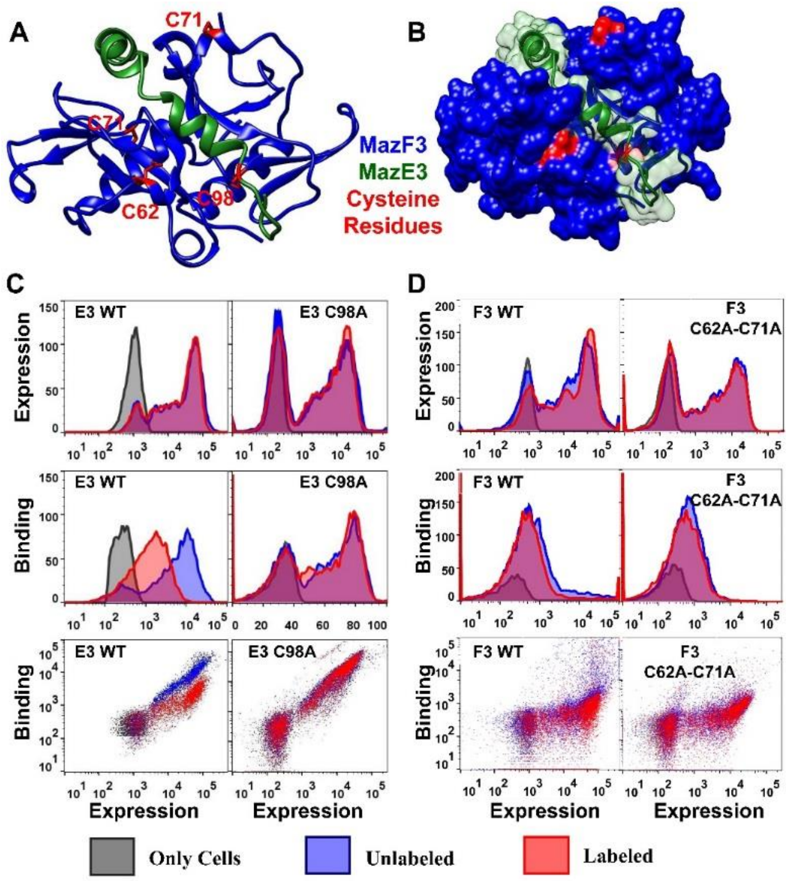

Figure S4 Effect of WT cysteine on binding after labeling in the MazEF3 system. (A) MazF3 dimers are shown in blue colour and the C-terminal region of the MazE antitoxin is shown in green colour. Structure is predicted from homology modelling. Residues C62 and C71 in MazF3 and residues C98 in MazE3 are highlighted in red colour. (B) Surface map showing that the residues C98 in MazE3 is close to the predicted interface. (C-D) Overlay of binding histograms of labeled and unlabeled cells shown in red and blue respectively. In surface displayed (C) WT MazE3 (the binding of cognate WT partner is reduced after cell surface labeling (left panels), indicating that the WT cysteine is part of the interface. However, upon mutation of the cysteine residue to alanine, in MazE3-C98A there is no change in binding after labeling. The cysteine library for MazE3 was thus constructed in the Cys→Ala background. Since, C62, C71 in MazF3 are not a part of the interface, the Ala mutants did not show any decrease in binding signal after labeling, however since MazF3 C62A-C71A, had much higher expression and binding signal as compared to the WT, the final MazF3 DMS library was made in the double mutant background.

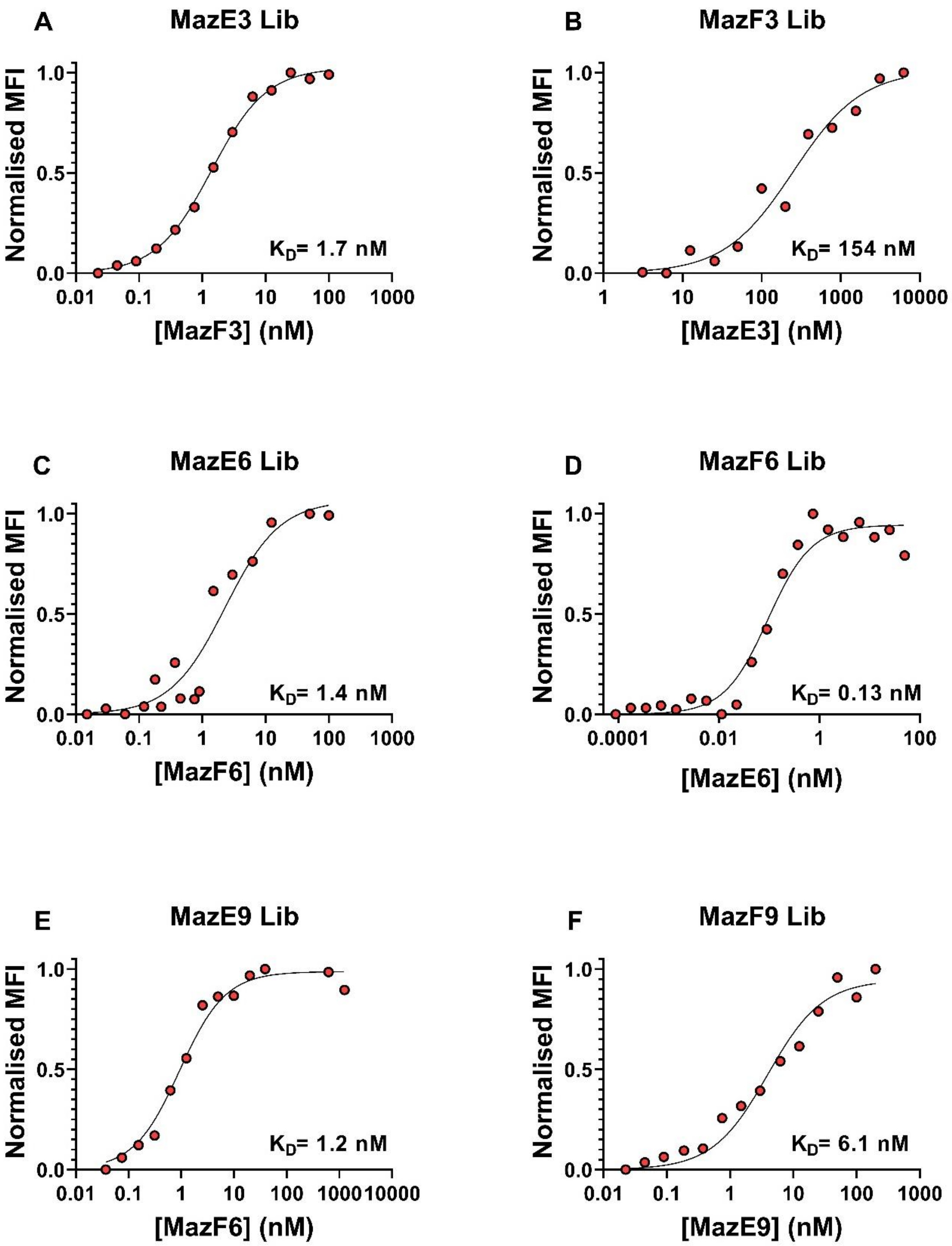

Figure S5 Apparent dissociation constants of the MazE and MazF cysteine libraries. Cysteine libraries were expressed on the yeast cell surface and incubated with different concentrations of the cognate partner. The amount of binding was measured with a BD Aria III cell sorter, employing the FLAG tag on the partner.

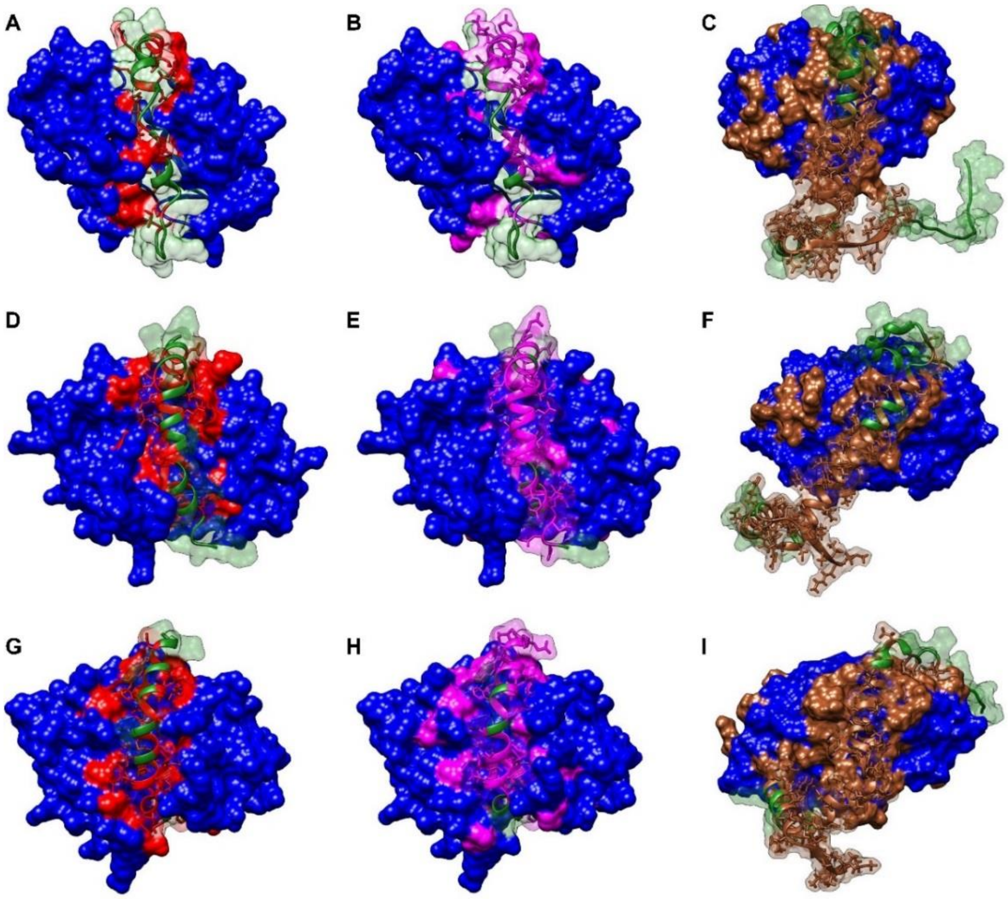

Figure S6 Comparison between interface residues predicted from the model and deep sequencing data for the three MazEF complexes. (A) The residues predicted to be a part of the interface of the MazEF3 complex (A) based on homology modelling which are highlighted in red, (B) experimentally verified residues from cysteine DMS libraries which are highlighted in magenta and (C) predicted interface residues from AlphaFold2 which are highlighted in brown. The residues predicted to be a part of the interface of the MazEF6 complex (D) based on homology modelling which are highlighted in red, (E) experimentally verified residues from cysteine DMS libraries which are highlighted in magenta and (F) predicted interface residues from AlphaFold2 which are highlighted in brown. (G) The residues predicted to be a part of the interface of the MazEF9 complex (G) based on homology modelling which are highlighted in red, (H) experimentally verified residues from cysteine DMS libraries which are highlighted in magenta and (I) predicted interface residues from AlphaFold2 which are highlighted in brown. The MazF toxins and the MazE antitoxins are coloured in blue (space filling) and green (ribbon) respectively.

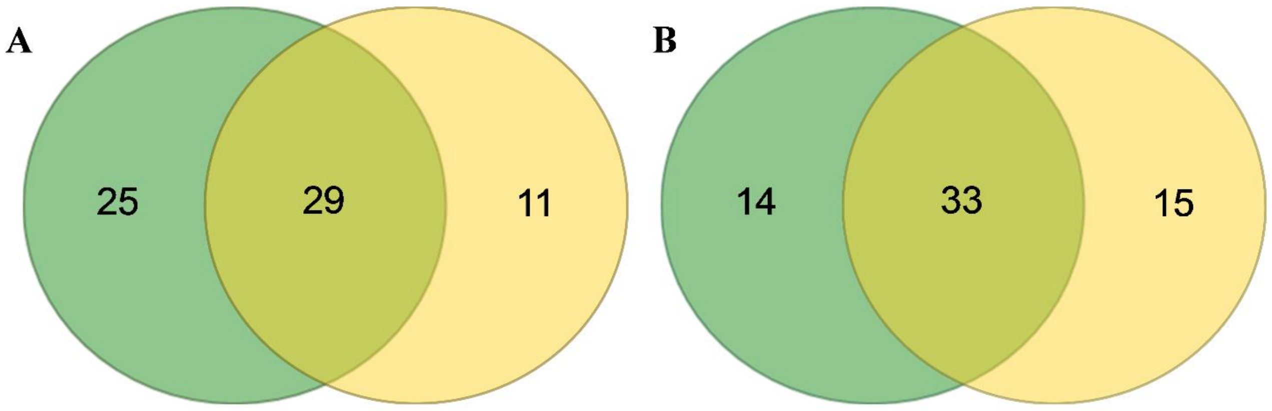

Figure S7 Comparison between interface residues predicted from the AlphaFold2 model and MazEF9 crystal structure. Venn diagram showing the overlap between the residues predicted to be a part of the interface from the AlphaFold2 model (green) and the MazEF9 crystal structure (yellow) for the (A) MazE9 antitoxin and the (B) MazF9 toxin.

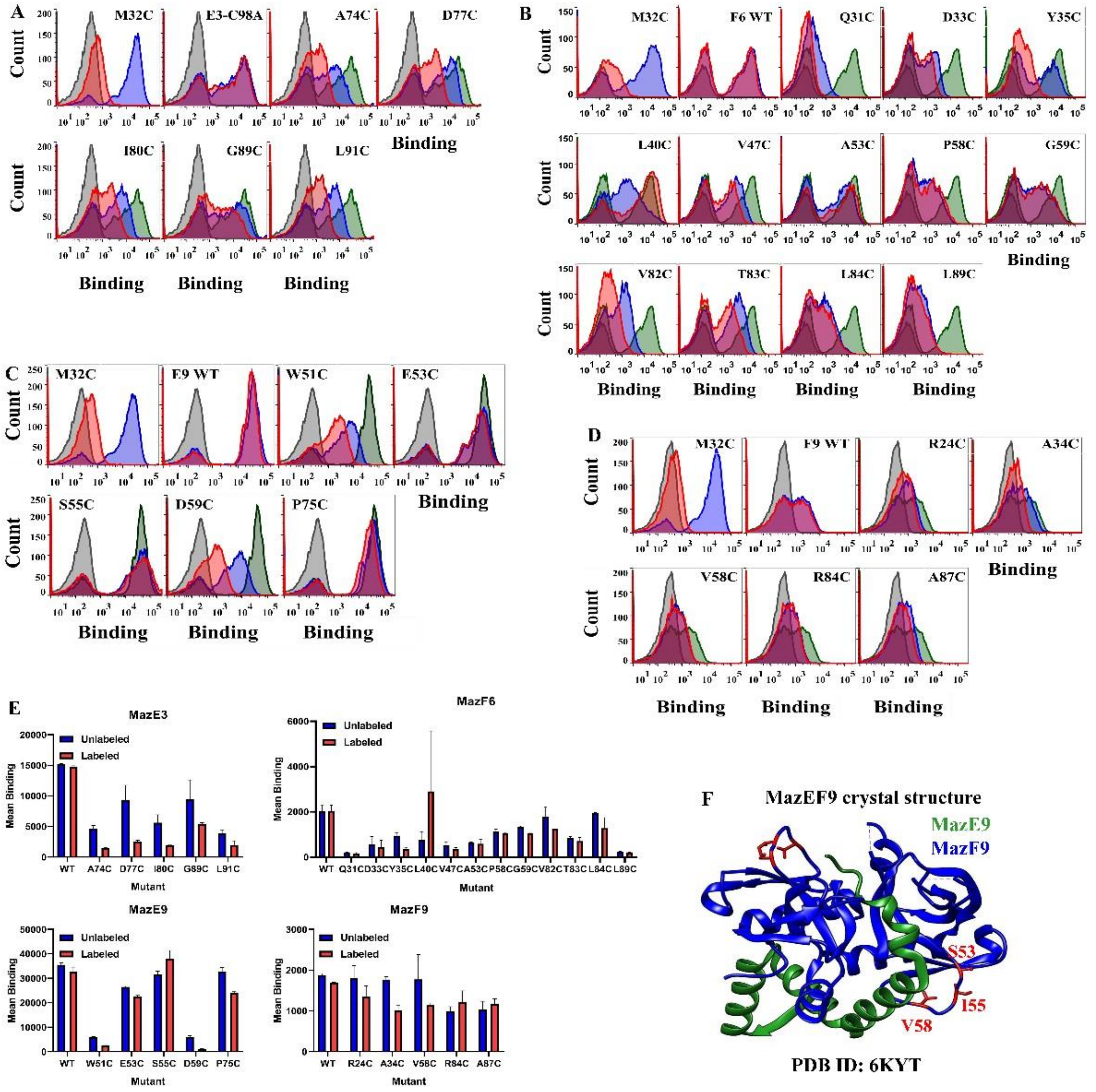

Figure S8 Overlay of binding histograms of individually analysed cysteine mutants. The mutants were selected from the deep sequencing data to validate predictions from analysis of FACS data from the MazE3-C98A, MazF6, MazE9 and MazF9 cysteine libraries. The mutants were selected to have different predicted levels of binding in the labeled and unlabeled cysteine mutants. Blue and red histograms for CcdB M32C (used as a labeling control, should show poor binding to its cognate partner GyrA14 upon labeling) and MazE3-C98A, MazF6, MazE9 and MazF9 WT represent unlabeled and labeled cells respectively and are identical for the MazE3-C98A, MazF6, MazE9 and MazF9 WT. The uninduced cells are shown in grey. For binding histogram overlays, green represents WT binding, blue and red are of unlabeled and labeled cysteine mutants respectively. (A) Cysteine mutants of probable

hot-spot residues for MazE3 (A74C, I80C and L91C) showed a large decrease in binding upon mutation. Mutants (D77C, and G89C) did not show much decrease in binding upon mutation but showed significantly reduced binding upon labeling. (B) Cysteine mutants of probable hot-spot residues for MazF6, Q31C, D33C, V47C, V82C, L84C and L89C showed a large decrease in binding upon mutation. Mutants L84C, L89C, P58C, G59C though showed reduction in binding upon mutation, but there was no further reduction upon labeling. Mutants Y35C and T83C did not show much decrease in binding upon mutation but showed significantly reduced binding upon labeling. The cysteine mutant L40C showed increased binding upon labeling, for reasons that are unclear. The mutant A53C did not show any change upon mutation, or mutation and labeling. (C) MazE9 cysteine mutants of probable hot-spot residues (W51C and D59C) showed a large decrease in binding upon mutation. The mutants E53C, S55C and P75C did not show any change upon mutation, or mutation and labeling. Of these, only E53C is part of the interface in the crystal structure. (D) MazF9 cysteine mutants R24C, R84C and A87C showed reduction in binding upon mutation but no further reduction upon labeling. The mutant A34C did not show much decrease in binding upon mutation but significantly reduced binding upon labeling. Surprisingly, a non-interacting mutant, based on the solved crystal structure of MazEF9 complex, V58C also showed reduction in binding upon mutation but no further reduction upon labeling. (E) The bar plots showing the Mean Binding values before and after labeling of the individual cysteine mutants. The error bars shown represent the standard deviation from two independent experiments, each performed in duplicates. (F) The *M. tuberculosis* MazEF9 crystal structure (PDB ID: 6KYT) showing the MazF9 cysteine mutants. The MazF9 mutants S53C, I55C and V58C although non-interacting were found to reduce the binding upon mutation from 1 D library sort. One possible justification for the reduction in binding upon mutation could be that these positions are present in a loop region of MazF9 toxin (shown in red colour in the MazEF9 crystal structure) and the cysteine mutations at these positions may result in increased aggregation of protein thereby showing a decrease in the binding signal.

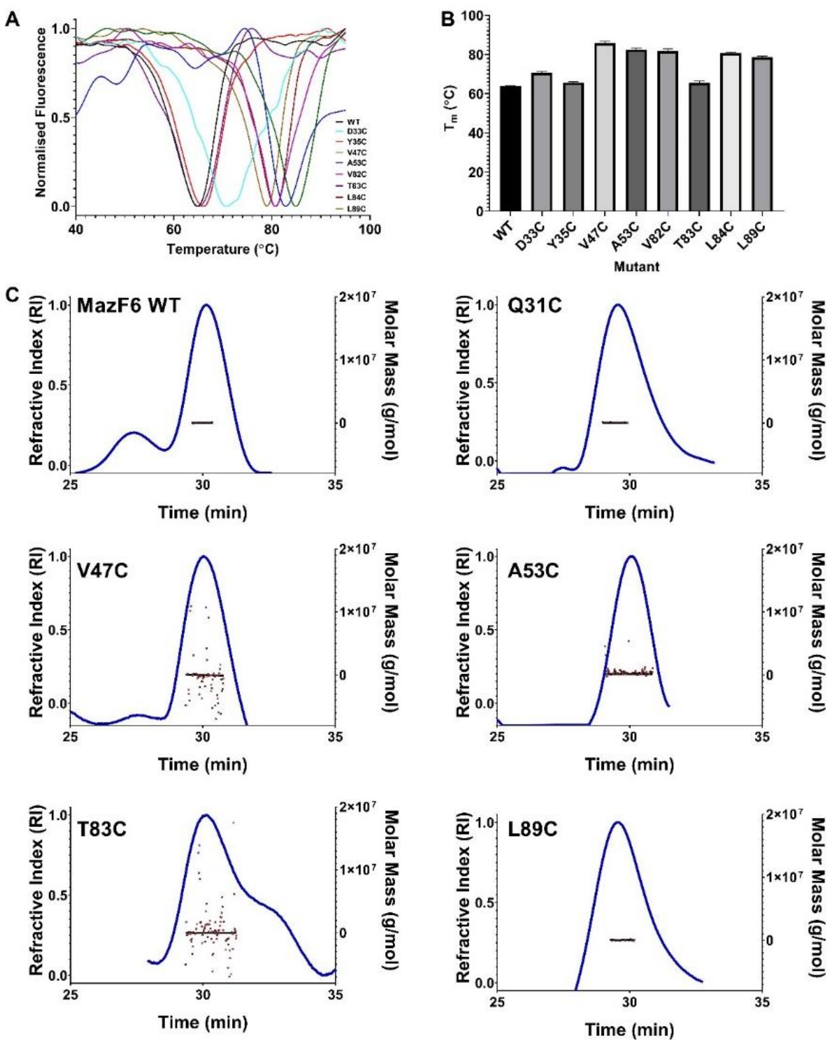

Figure S9 Biophysical characterisation of purified MazF6 cysteine mutants. (A-B) Thermal stabilities of purified MazF6 cysteine mutants. (A) Thermal unfolding profile of 5 µM of purified WT MazF6 (black), D33C (cyan), Y35C (red), V47C (green), A53C (blue), V82C (magenta), T83C (violet), L84C (maroon) and L89C (brown) carried out by nanoDSF. (B) Thermal stability, T_m of the different MazF6 cysteine mutants. The error bars shown represent the standard deviation from two independent experiments, each performed in duplicates. (C) Oligomeric analysis of purified MazF6 cysteine mutants by SEC MALS. Traces for refractive index are shown in blue. The molar mass and fits of all traces are plotted as a function of elution time as approximately horizontal red and black lines respectively. The peaks analysed for molecular weight determination are shown in each graph for MazF6 WT, MazF6 Q31C, MazF6 V47C, MazF6 A53C, MazF6 T83C and MazF6 L89C. The molar mass and mass fraction of each peaks is listed in Table S11.

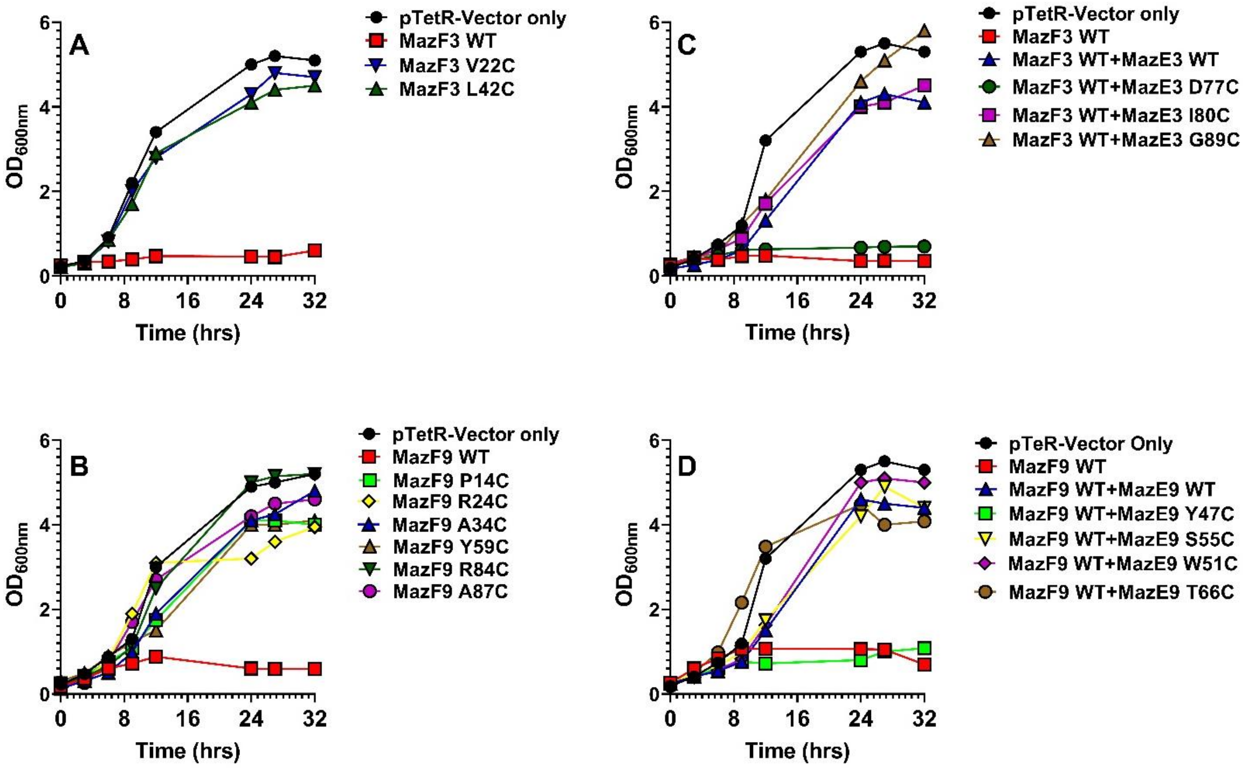

Figure S10 ***In vivo*** **phenotypes of cysteine mutant upon overexpression in** ***M. smegmatis*****.** The effect of overexpression of MazF toxins and MazE6 antitoxin on mycobacterial growth was assayed by measuring OD_{600nm} after induction with 50 ng/ml of anhydrotetracycline (Atc) and 0.2% of acetamide respectively. (A-B) *Mycobacterium tuberculosis* H37Rv strain was transformed either with Atc inducible vector alone or mazF toxins by electroporation. Compared with the strain harbouring only vector, overexpression of MazF3 and MazF9 toxins reduced the bacterial loads by the 7th day after induction. The overexpression of the cysteine mutants at residues inferred to be at the MazEF interface of MazF3 and MazF9 did not inhibit *Mycobacterium smegmatis* growth. (C-D) In the co-expression experiment, the expression of the WT MazE3, WT MazE9 and all the MazE3 and MazE9 cysteine antitoxin mutants (except for MazE3-D77C and MazE9-Y47C) were able to neutralise the activity of the WT cognate MazF3 and MazF9 toxins respectively. The data presented are representative of three independent biological replicates.

Supplementary Tables

Table S1. Templates used for modelling of different MazEF toxins and antitoxins.

**TA system**	**Protein**	**Organism**	**Template identified by Hhpred**	**Percentage identity**	**Percentage query coverage**	**E-value**
MazEF3	MazF3	*B. subtilis*	4mdx	26	97	5.00E-030
	MazE3	*B. subtilis*	4me7	17	68	4.00E-011
MazEF6	MazF6	*B. subtilis*	4mdx	38	98	9.00E-036
	MazE6	*B. subtilis*	4me7	18	93	4.60E-015
MazEF9	MazF9	*B. subtilis*	4mdx	34	96	1.60E-033
	MazE9	*B. subtilis*	4me7	21	86	4.60E-015

Table S2. The mass fractions and corresponding molecular weights of each of the peaks for the proteins of the MazEF TA system analysed by SEC-MALS (T:Toxin, A:Antitoxin).

Proteins	Peak	Observed molecular weight (kDa)	Expected molecular weight (kDa)	Radius of gyration, R_g (nm)	Mass fraction	Stoichiometry
MazE3	1<br>2	33.6<br>751.4	33.8	23±7	36.5<br>63.5	A_2 (Dimer)<br>Aggregate
MazF3	1<br>2	33.8<br>1491	33.2	27±4	70.2<br>29.8	T_2 (Dimer)<br>Aggregate
MazEF3	1<br>2	77.6<br>1768	73.4	156±88	14.5<br>85.5	$T_2A_2T_2$ (Hetero-hexamer)<br>Aggregate
MazF3+excess MazE3 peptide	1<br>2	43.8<br>2111	40.5	54±7	93.0<br>7.0	AT-TA (Hetero-Tetramer)<br>Aggregate
MazE9	1	26.9	27.6	29±11	100	A_2 (Dimer)
MazF9	1<br>2	36.2<br>848.3	36.8	32±6	98.8<br>1.2	T_2 (Dimer)<br>Aggregate
MazEF9	1<br>2<br>3	30.5<br>84.7<br>1470	82.0	117±21	14.3<br>77.2<br>8.5	T_2/A_2 (Dimer)<br>$T_2A_2T_2$ (Hetero-hexamer)<br>Aggregate
MazF9+excess MazE9 peptide	1<br>2	43.2<br>446.3	44.2	46±7	87.0<br>13.0	AT-TA (Hetero-Tetramer)<br>Aggregate

Table S3. Summary of the k-means clustering parameters used to identify interacting residues.

Class 1 and Class 2 refer to mutant with WT like or lower binding affinity than WT respectively.

Library	Class 1 (Mean)	Class 1 (Std. Dev)	Class 2 (Mean)	Class 2 (Std. Dev)	Criteria for selection	Cut-off used for $MFI_{depletion}^{Mutant}$
MazE3 pooled	0.75	0.23	1.70	0.22	$mean^{Class1} + 2\sigma^{class1}$	≥1.2
MazF3 pooled	0.76	0.33	3.20	2.1	$mean^{Class1} + 1.4\sigma^{class1}$	≥1.2
MazE6 pooled	0.74	0.28	1.80	0.56	$mean^{Class1} + 1.5\sigma^{class1}$	≥1.2
MazF6 pooled	0.99	0.25	4.69	3.55	$mean^{Class1} + 1\sigma^{class1}$	≥1.2
MazE9 pooled	1.10	0.23	2.32	1.40	$mean^{Class1} + 0.5\sigma^{class1}$	≥1.2
MazF9 pooled	0.32	0.32	3.87	4.40	$mean^{Class1} + 2.5\sigma^{class1}$	≥1.2

Table S4. List of mutants of MazE3 selected for cysteine scanning mutagenesis, and interface residues in MazEF3 AlphaFold2 or homology models, or inferred from Cys scanning. In the first and second columns, the (┼) sign indicates interface mutants selected based on the MazEF3 AlphaFold2 and homology models respectively. The third column is the experimentally identified mutants of the MazE3 cysteine library from the vertical gate. The (┼) sign in magenta represents reduction in binding upon mutation and labeling. The orange represents reduced binding (≥15%) upon mutation, but no further reduction was observed upon labeling. The black sign represents no change in binding upon mutation or labeling. Red represent no change in binding after mutation but increase in binding after labeling (≥1.2 fold).

**Mutants**	**Predicted interacting residues from AlphaFold2 model**	**Predicted interacting residues from the homology model**	**Identified from DMS-FACS**
**I26C**	┼		-
**E28C**	┼		-
**V29C**	┼		-
**S30C**	┼		-
**T31C**	┼		-
**Q32C**	┼		-
**I33C**	┼		-
**A34C**	┼		-
**V35C**	┼		-
**R36C**	┼		-
**L37C**	┼		-
**P38C**	┼		-
**D39C**	┼		-
**E40C**	┼		-
**I41C**	┼		-
**V42C**	┼		-
**F44C**	┼		-
**I45C**	┼		-
**E48C**	┼		-
**H53C**	┼		-
**R57C**	┼		-
**A58C**	┼		-
**V60C**	┼		-

**V61C**	+		-
**L62C**	+		-
**R63C**	+		-
**A64C**	+		-
**L65C**	+		-
**E66C**	+		-
**R67C**	+		-
**E68C**	+		-
**R69C**	+		-
**R70C**	+		-
**R71C**	+		-
**R72C**	+		+
**L73C**	+	+	+
**A74C**	+	+	+
**E75C**	+		+
**R76C**	+		*
**D77C**	+	+	+
**A78C**	+	+	+
**E79C**			+
**I80C**	+		+
**L81C**	+	+	+
**A82C**	+		+
**T83C**			+
**N84C**	+	+	+
**T85C**	+		+
**S86C**			+
**A87C**	+		+
**T88C**		+	+
**G89C**	+		+
**D90C**	+	+	*
**L91C**	+	+	*
**D92C**	+	+	*
**T93C**	+		+
**L94C**	+		*
**A95C**	+	+	*
**G96C**	+		+
**H97C**	+		*
**A98C**	+	+	+
**A99C**	+		*
**R100C**	+		*
**T101C**	+		*

**A102C**	+		+
**L103C**	+		*
**D104C**	+		*
**I105C**	+	+	*
**D106C**	+		*

-: Not chosen for cysteine mutagenesis

*: No read counts associated with this mutant or total read count less than the cut-off used.

Table S5. List of mutants of MazF3 selected for cysteine scanning mutagenesis, and interface residues in MazEF3 AlphaFold2 or homology models, or inferred from Cys scanning. In the first and second columns, the (+) sign indicates interface mutants selected based on the MazEF3 AlphaFold2 and homology models respectively. The third column is the experimentally identified mutants of the MazF3 cysteine library from the vertical gate. The (+) sign in magenta represents reduction in binding upon mutation and labeling. The orange represents reduced binding (≥15%) upon mutation, but no further reduction was observed upon labeling. The black sign represents no change in binding upon mutation or labeling. Red represent no change in binding after mutation but increase in binding after labeling (≥1.2 fold).

Mutants	Predicted interacting residues from AlphaFold2	Predicted interacting residues from the homology model	Identified from the DMS-FACS
M1C	+	+	-
P3C		+	-
Q8C	+		-
L9C	+		-
D10C	+		-
K11C	+		-
R13C	+		+
R20C	+		+
E21C	+		-
V22C	+	+	*
V23C	+		+
R24C	+		-
P25C	+		-
H26C	+	+	+
L27C	+	+	+
T28C	+	+	+
N29C	+	+	+
P34C	+		+
T36C	+		-
T37C	+		-
T38C	+		*

**V39C**	+		+
**R40C**	+		-
**G41C**			+
**L42C**	+	+	+
**A43C**	+		+
**V51C**	+		-
**S61C**	+		-
**A62C**			+
**D63C**	+	+	-
**N64C**	+	+	-
**Q66C**	+	+	*
**T67C**	+	+	*
**I68C**	+		-
**P69C**	+	+	*
**D72C**	+	+	*
**Q76C**	+		-
**Y79C**	+		-
**L81C**	+		-
**A82C**	+		-
**E90C**	+		-
**N94C**	+		-
**D97C**	+		-
**D99C**	+		-
**V101C**	+		-
**V102C**	+		-
**A103C**	+		-

-: Not chosen for cysteine mutagenesis

*: No read counts associated with this mutant or total read count less than the cut-off used.

Table S6. List of mutants of MazE6 selected for cysteine scanning mutagenesis, and interface residues in MazEF6 AlphaFold2 or homology models, or inferred from Cys scanning. In the first and second columns, the (┼) sign indicates interface mutants selected based on the MazEF6 AlphaFold2 and homology models respectively. The third column is the experimentally identified mutants of the MazE6 cysteine library from the vertical gate. The (┼) sign in magenta represents reduction in binding upon mutation and labeling. The orange represents reduced binding (≥15%) upon mutation, but no further reduction was observed upon labeling. The black sign represents no change in binding upon mutation or labeling. Red represent no change in binding after mutation but increase in binding after labeling (≥1.2 fold).

Mutants	Predicted interacting residues from AlphaFold2	Predicted interacting residues from the homology model	Identified from the DMS-FACS
**M1C**	┼		-
**K2C**	┼		-
**T3C**	┼		-
**A4C**	┼		-
**I5C**	┼		-
**S6C**	┼		-
**L7C**	┼		-
**P8C**	┼		-
**D9C**	┼		-
**T11C**	┼		-
**F12C**	┼		-
**D13C**	┼		-
**R14C**	┼		-
**V15C**	┼		-
**R18C**	┼		-
**E21C**	┼		-
**L22C**	┼		-
**M24C**	┼		-
**R26C**	┼		-
**S27C**	┼		-
**F29C**	┼		-

**F30C**	+		-
**T31C**	+		-
**K32C**	+		-
**A33C**	+		-
**A34C**	+		-
**Q35C**	+		-
**R36C**	+		-
**Y37C**	+		-
**L38C**	+		-
**H39C**	+		-
**E40C**	+		-
**L41C**	+		-
**D42C**	+		-
**A43C**	+		-
**Q44C**	+		-
**L45C**	+		-
**L46C**	+		-
**T47C**	+		-
**G48C**			+
**Q49C**	+		+
**I50C**	+	+	+
**D51C**	+	+	+
**R52C**			+
**A53C**	+	+	*
**L54C**	+	+	+
**E55C**	+		+
**S56C**	+		+
**I57C**	+	+	+
**H58C**		+	+
**G59C**		+	+
**T60C**	+		*
**D61C**	+		+
**E62C**		+	+
**A63C**		+	+
**E64C**		+	+
**A65C**			+
**L66C**		+	+
**A67C**			+
**V68C**			+
**A69C**		+	+

**N70C**		+	+
**A71C**			+
**Y72C**			+
**R73C**		+	+
**V74C**		+	+
**L75C**			+
**E76C**			+
**T77C**		+	+
**M78C**			+
**D79C**			+
**D80C**			+
**E81C**			+
**W82C**	+		*

-: Not chosen for cysteine mutagenesis

*: No read counts associated with this mutant or total read count less than the cut-off used.

Table S7. List of mutants of MazF6 selected for cysteine scanning mutagenesis, and interface residues in MazEF6 AlphaFold2 or homology models, or inferred from Cys scanning. In the first and second columns, the (+) sign indicates interface mutants selected based on the MazEF6 AlphaFold2 and homology models respectively. The third column is the experimentally identified mutants of the MazF6 cysteine library from the vertical gate. The (+) sign in magenta represents reduction in binding upon mutation and labeling. The orange represents reduced binding (≥15%) upon mutation, but no further reduction was observed upon labeling. The black sign represents no change in binding upon mutation or labeling. Red represent no change in binding after mutation but increase in binding after labeling (≥1.2 fold).

Mutants	Predicted interacting residues from AlphaFold2	Predicted interacting residues from the homology model	Identified from DMS-FACS
**M1C**	+		-
**V2C**	+		-
**S4C**	+		-
**E7C**	+		-
**A11C**		+	-
**L13C**		+	-
**G14C**		+	-
**R25C**		+	*
**P26C**			-
**Q31C**			+
**D33C**			+
**P34C**	+	+	-
**Y35C**	+	+	+
**A37C**	+		+
**S38C**	+	+	-
**R39C**	+	+	-
**L40C**	+	+	+
**A41C**		+	+
**T42C**		+	*
**V47C**			+
**T49C**		+	-

**N51C**	+	+	*
**A53C**	+		+
**L54C**	+		-
**A56C**	+	+	*
**M57C**	+	+	*
**P58C**	+		+
**G59C**			+
**N60C**		+	-
**R69C**	+		-
**N77C**		+	-
**T79C**		+	-
**A80C**		+	-
**I81C**			*
**V82C**		+	+
**T83C**		+	+
**L84C**		+	+
**N85C**		+	+
**D88C**			-
**L89C**			+
**R92C**	+		-
**E95C**	+		-
**R110C**	+		-
**D113C**	+		-

-: Not chosen for cysteine mutagenesis

*: No read counts associated with this mutant or total read count less than the cut-off used.

Table S8. List of mutants of MazE9 selected for cysteine scanning mutagenesis, and interface residues either identified from the MazEF9 crystal structure or from the DMS-FACS experimental data. In the first and second columns, the (┼) sign indicates interface mutants selected based on the MazEF9 AlphaFold2 and homology models respectively. The third column is the experimentally identified mutants of the MazE9 cysteine library from FACS. The fifth column shows the corresponding ΔASA values from the MazEF9 crystal structure (PDB ID: 6KYT). The (┼) sign in magenta represents reduction in binding upon mutation and labeling. The orange represents reduced binding (≥15%) upon mutation, but no further reduction was observed upon labeling. The black sign represents no change in binding upon mutation or labeling. Red represent increase in binding after labeling (≥1.2 fold).

**Mutants**	**Predicted interacting residues from AlphaFold2 model**	**Predicted interacting residues from the homology model**	**Identified from DMS-FACS**	**ΔASA (Å^2)**
**M1C**	┼		-	0.0
**K2C**	┼		-	0.0
**L3C**	┼		-	0.0
**S4C**	┼		-	0.0
**V5C**	┼		-	0.0
**S6C**	┼		-	0.0
**L7C**	┼		-	0.0
**S8C**	┼		-	0.0
**D9C**	┼		-	0.0
**D10C**	┼		-	0.0
**D11C**	┼		-	0.0
**V12C**	┼		-	0.0
**I14C**	┼		-	0.0
**L15C**	┼		-	0.0
**Y18C**	┼		-	3.0
**R21C**	┼		-	4.4
**A22C**	┼		-	10.0
**G23C**	┼		-	77.8
**L24C**			-	15.6
**P25C**	┼		-	33.0
**R27C**	┼		-	0.0

**S28C**	+		-	0.0
**A29C**			-	20.1
**L31C**	+		-	0.0
**Q32C**	+		-	6.1
**H33C**	+		-	30.6
**A34C**	+		-	0.0
**I35C**	+		-	0.0
**R36C**	+		-	53.1
**V37C**	+		-	26.3
**L38C**	+		-	0.0
**R39C**	+		-	0.0
**Y40C**	+		-	26.4
**P41C**	+		-	1.4
**T42C**	+		-	0.0
**L43C**	+		+	50.3
**E44C**	+		+	56.4
**D45C**	+		+	2.7
**D46C**	+	+	+	43.9
**Y47C**	+	+	+	62.7
**A48C**	+	+	*	45.6
**N49C**	+		+	17.4
**A50C**	+	+	+	68.3
**W51C**	+	+	+	70.9
**Q52C**	+	+	+	9.9
**E53C**	+		+	62
**W54C**	+	+	+	48.8
**S55C**	+	+	+	0.0
**A56C**			*	0.0
**A57C**	+		*	0.0
**G58C**		+	+	0.0
**D59C**	+	+	+	29.8
**T60C**			+	30.6
**D61C**	+	+	+	0.0
**A62C**		+	+	56.1
**W63C**	+	+	+	73.8
**E64C**		+	+	38.1
**Q65C**		+	+	62.6
**T66C**	+	+	+	55.0
**V67C**	+	+	*	83.5
**G68C**	+	+	*	78.6
**D69C**		+	*	64.9

**G70C**	+		*	107.9
**V71C**		+	*	83.3
**G72C**			*	84.3
**D73C**			*	0.0
**A74C**		+	*	0.0
**P75C**		+	+	0.0
**R76C**		+	*	0.0

-: Not chosen for cysteine mutagenesis

*: No read counts associated with this mutant or total read count less than the cut-off used.

Table S9. List of mutants of MazF9 selected for cysteine scanning mutagenesis, and interface residues either identified from the MazEF9 crystal structure or from the DMS-FACS experimental data. In the first and second columns, the (+) sign indicates interface mutants selected based on the MazEF9 AlphaFold2 and homology models respectively. The third column is the experimentally identified mutants of the MazF9 cysteine library from the FACS. The fifth column shows the corresponding ΔASA values from the MazEF9 crystal structure (PDB ID: 6KYT). The (+) sign in magenta represents reduction in binding upon mutation and labeling. The orange represents reduced binding (≥15%) upon mutation, but no further reduction was observed upon labeling. The black sign represents no change in binding upon mutation or labeling. Red represent no change increase in binding after labeling (≥1.2 fold).

Mutants	Predicted interacting residues from AlphaFold2 model	Predicted interacting residues from the homology model	Identified from DMS-FACS	ΔASA (Å^2)
R3C	+		-	0.0
Q9C	+		-	0.0
V10C			-	4.3
L12C		+	*	42.3
D13C			*	0.0
P14C			*	26.8
S18C	+	+	-	0.0
E19C		+	-	0.0
A20C	+	+	-	0.0
N21C	+	+	-	0.6
N22C	+	+	-	41.7
Q23C	+		-	0.0
R24C	+	+	+	39.5
P25C	+		-	0.0
A26C			-	6.8
N31C	+	+	-	0.0
D32C	+		+	0.0
R33C	+	+	+	81.7

**A34C**	+	+	+	40.1
**Y36C**	+		-	4.6
**T37C**	+	+	*	53.2
**A38C**	+		-	5.8
**R40C**	+		-	9.5
**L41C**	+		*	18.0
**G42C**		+	+	0.0
**R43C**	+	+	-	6.3
**G44C**	+		-	49.4
**V45C**	+		*	27.0
**P50C**		+	*	3.9
**I51C**		+	-	0.0
**T52C**	+	+	*	7.1
**S53C**	+		+	0.0
**N54C**	+	+	-	23.5
**I55C**		+	+	0.0
**A56C**	+		-	3.1
**K57C**	+		*	3.7
**V58C**			+	0.0
**Y59C**	+	+	+	45.1
**P60C**	+	+	+	51.1
**F61C**	+	+	*	39.4
**Q62C**	+	+	-	4.1
**L72C**	+		-	0.0
**Q73C**	+		-	0.0
**V74C**	+		-	0.0
**C76**	+	+	-	0.0
**K77C**	+	+	-	8.4
**A78C**		+	-	0.0
**Q79C**	+	+	-	24.2
**D81C**	+	+	-	19.4
**Q82C**	+	+	-	27.3
**I83C**				0.0
**R84C**		+	+	35.7
**S85C**		+	+	0.0
**I86C**			*	17.0
**A87C**			+	32.6
**R90C**			*	22.9
**R93C**	+		-	0.0
**R97C**	+		-	7.3

**A100C**	┼		-	55.2
**E108C**	┼		-	1.7
**L112C**	┼		-	52.9
**H113C**	┼		-	3.5
**D115C**	┼		-	34.2
**W117C**	┼		-	44.2
**S118C**	┼		-	6.0

-: Not chosen for cysteine mutagenesis

*: No read counts associated with this mutant or total read count less than the cut-off used.

Table S10. The mass fractions and corresponding molecular weights for the proteins of the MazF6 WT and cysteine mutants analysed by SEC-MALS.

Protein	Observed molecular weight (kDa)	Expected molecular weight (kDa)	Mass fraction	Stoichiometry
MazF6 WT	32.0	35.52	81.0	T_2 (Dimer)
Q31C	31.4	35.47	74.3	T_2 (Dimer)
V47C	29.6	35.53	96.4	T_2 (Dimer)
A53C	27.1	35.59	53.0	T_2 (Dimer)
T83C	28.6	35.53	64.1	T_2 (Dimer)
L89C	31.0	35.50	92.7	T_2 (Dimer)

References

Chattopadhyay, G., Bhasin, M., Ahmed, S., Gosain, T. P., Ganesan, S., Das, S., et al. (2022). Functional and Biochemical Characterization of the MazEF6 Toxin-Antitoxin System of Mycobacterium tuberculosis. *J. Bacteriol.* 204, e0005822. doi: 10.1128/jb.00058-22.

Jerabek-Willemsen, M., Wienken, C. J., Braun, D., Baaske, P., and Duhr, S. (2011). Molecular interaction studies using microscale thermophoresis. *Assay Drug Dev. Technol.* 9, 342–353. doi: 10.1089/adt.2011.0380.

Seidel, S. A. I., Wienken, C. J., Geissler, S., Jerabek-Willemsen, M., Duhr, S., Reiter, A., et al. (2012). Label-free microscale thermophoresis discriminates sites and affinity of protein-ligand binding. *Angew. Chemie - Int. Ed.* 51, 10656–10659. doi: 10.1002/anie.201204268.

Wienken, C. J., Baaske, P., Rothbauer, U., Braun, D., and Duhr, S. (2010). Protein-binding assays in biological liquids using microscale thermophoresis. *Nat. Commun.* 1, 1038. doi: 10.1038/ncomms1093.
